# Engineering Nanoemulsions to Maximize NIR‐II Fluorescence and Preserve Photothermal Performance of a Novel Boron Difluoride Formazanate Dye

**DOI:** 10.1002/anie.4378015

**Published:** 2026-05-04

**Authors:** Nahyun Kwon, Francis L. Buguis, Theo Husby, Suhjung Chun, Dongling Zhang, Jiaze Wu, Benjamin Rehl, Binbing Ling, Umar Iqbal, Melissa Washington, Angie Verner, Kai Huang, Juan Chen, Joe B. Gilroy, Gang Zheng

**Affiliations:** ^1^ Princess Margaret Cancer Centre University Health Network Toronto Canada; ^2^ Department of Chemistry The University of Western Ontario London Canada; ^3^ Department of Medical Biophysics University of Toronto Toronto Canada; ^4^ Human Health Therapeutics Research Center National Research Council Canada Ottawa Canada; ^5^ Department of Materials Science and Engineering University of Toronto Toronto Canada; ^6^ Department of Electrical and Computer Engineering University of Toronto Toronto Canada

**Keywords:** boron difluoride formazanate, nanoemulsion, NIR‐II fluorescence imaging, photoacoustic imaging, photothermal therapy

## Abstract

Boron difluoride formazanate (BDF) dyes possess intrinsic NIR‐I absorption and NIR‐II photoluminescence. However, their hydrophobic nature often leads to fluorescence quenching in polar aqueous environment, limiting their performance in biological applications. Here, we report a newly synthesized BDF dye (**3**) formulated as an oil‐in‐water nanoemulsion (**BDF‐NE**) that overcomes this challenge by providing a nonpolar oil core microenvironment that closely matches the favorable conditions required to preserve the bright emission of **3**. Molecular solubilization of **3** within a glyceryl trioctanoate core, stabilized by a phospholipid/PEG‐lipid shell, maintains strong NIR absorption and a high molar extinction coefficient while simultaneously maximizing NIR‐II emission intensity. **BDF‐NE** achieves a photothermal conversion efficiency of 66.8%, generates strong photoacoustic (PA) contrast at 780 nm, and exhibits bright NIR‐II fluorescence extending beyond 1250 nm with an absolute quantum yield of 2.9%, enabling high‐resolution vascular imaging and real‐time tracking of tumor accumulation in vivo. In subcutaneous tumor‐bearing mice, dual‐modal NIR‐II/PA imaging‐guided photothermal therapy achieves complete ablation of tumors in a subset of mice and significantly prolongs recurrence‐free survival without detectable systemic toxicity. This nanoemulsion‐based strategy unlocks the full dual‐modal theranostic potential inherent to BDF dyes and offers a generalizable strategy for translating hydrophobic NIR fluorophores into high‐performance theranostic agents.

## Graphical abstract

An oil‐in‐water nanoemulsion transforms a boron difluoride formazanate (BDF) dye into a dual‐modal imaging‐guided theranostic agent (BDF‐NE). Its nonpolar oil core maximizes NIR‐II fluorescence while maintaining strong photoacoustic contrast to guide photothermal therapy with high photostability and 66.8% photothermal conversion efficiency, achieving complete tumor ablation in vivo and highlighting formulation‐driven performance enhancement.

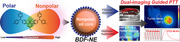

## 1 Introduction

Photothermal therapy (PTT), which relies on the conversion of light energy into heat to induce localized tumor ablation, offers several notable advantages over conventional cancer treatments, including high spatial precision, minimal invasiveness, and reduced systemic toxicity [1, 2, 3]. When paired with real‐time imaging, PTT can be performed with exceptional spatiotemporal control, enabling accurate treatment planning, and on‐demand therapeutic interventions [1, 4, 5]. Among available imaging modalities, photoacoustic (PA) imaging is particularly well‐suited for guiding PTT, as PA signals arise from thermoelastic expansion following optical absorption, making the technique intrinsically compatible with photothermal processes [6, 7]. By combining optical excitation with ultrasound detection, PA imaging provides high resolution optical contrast at depths that exceed the limit of conventional optical imaging [8].

Second near‐infrared (NIR‐II, 1000–1700 nm) fluorescence imaging offers a highly complementary set of advantages, including reduced photon scattering, minimal tissue autofluorescence, and enhanced penetration depth compared to NIR‐I (700–900 nm) fluorescence [9, 10]. Considerable efforts have therefore been devoted to developing bright, stable NIR‐II fluorophores for biomedical imaging and theranostic applications. This need is further underscored by the limitation of indocyanine green (ICG), the only FDA‐approved NIR fluorophore, which emits into the NIR‐II region only through a weak tail of its NIR‐I emission, rather than through intrinsic NIR‐II fluorescence [11, 12]. Nevertheless, fluorescence imaging alone often lacks the anatomical context and depth localization necessary for precise treatment planning in heterogeneous tumor microenvironments. The complementary capabilities of NIR‐II fluorescence and PA imaging therefore motivate the development of synergistic dual‐model platform that unite the high molecular sensitivity of NIR‐II fluorescence with the deep‐tissue spatial resolution of PA imaging for accurate image‐guided PTT.

Boron difluoride formazanate (BDF) dyes have recently emerged as promising candidates for NIR‐II imaging due to their unique electronic structure [13, 14, 15]. In 2018, the Gilroy group demonstrated that a simple BDF derivative bearing N,N‐dimethylaniline (MW = 383 g/mol) exhibits absorption at 728 nm and photoluminescence at 888 nm, with an emission tail extending into the NIR‐II window, challenging the prevailing assumption that NIR‐II emitters require extensively conjugated architectures [16]. Subsequent mechanistic investigations revealed that the long‐wavelength emission in the BDF family arises from the pronounced quinoidal character of the *N*‐aryl substituents, which facilitates efficient electronic delocalization without the need for extended π‐conjugation [17, 18]. Building on this discovery, significant efforts have sought to translate BDF dyes into theranostic platforms [19, 20, 21, 22, 23, 24, 25, 26, 27]. Most studies have focused on photothermal therapy applications, reflecting a practical limitation: amine‐substituted BDF dyes are highly sensitive to solvent polarity, with fluorescence quenched even in moderately polar organic solvents [17]. In aqueous biological environments, this polarity‐induced quenching, often compounded by aggregation of the hydrophobic dye, renders fluorescence‐based imaging impractical. As a result, although BDF dyes perform effectively as photothermal agents, their intrinsic NIR‐II emissive properties remain largely unexploited, limiting the realization of their full dual‐modal theranostic potential. Preserving NIR‐II photophysical properties of BDF dyes in aqueous media thus represent a major unmet challenge.

Herein, we report a newly synthesized BDF dye (**3**) and its oil‐in‐water nanoemulsion formulation (**BDF‐NE**), designed to overcome this limitation. By leveraging the polarity‐dependent fluorescence modulation of BDF dyes, we engineered a controlled nonpolar microenvironment through molecular solubilization of **3** within a glyceryl trioctanoate core, stabilized by a phospholipid/PEG‐lipid shell. This formulation preserves the NIR absorption profile and high molar extinction coefficient of **3** while maximizing NIR‐II emission intensity. **BDF‐NE** achieves a photothermal conversion efficiency of 66.8%, generates strong PA contrast near 780 nm, and emits bright NIR‐II fluorescence beyond 1250 nm with an absolute quantum yield of 2.9% (1000‒1800 nm), collectively enabling high performance dual‐modal NIR‐II fluorescence and PA imaging to guide PTT. In KB tumor‐bearing mice, **BDF‐NE**‐mediated PTT achieves complete tumor ablation in a subset of animals and significantly prolongs recurrence‐free survival without detectable systemic toxicity. These findings establish nonpolar microenvironment engineering via nanoemulsion encapsulation as a versatile and generalizable strategy for diverse BDF dyes, unlocking their full dual‐modal theranostic potential in aqueous biological environments.

## 2 Results and Discussion

### 2.1 Synthesis and Photophysical Properties of Boron Difluoride Formazanate 3

**FIGURE 1:**
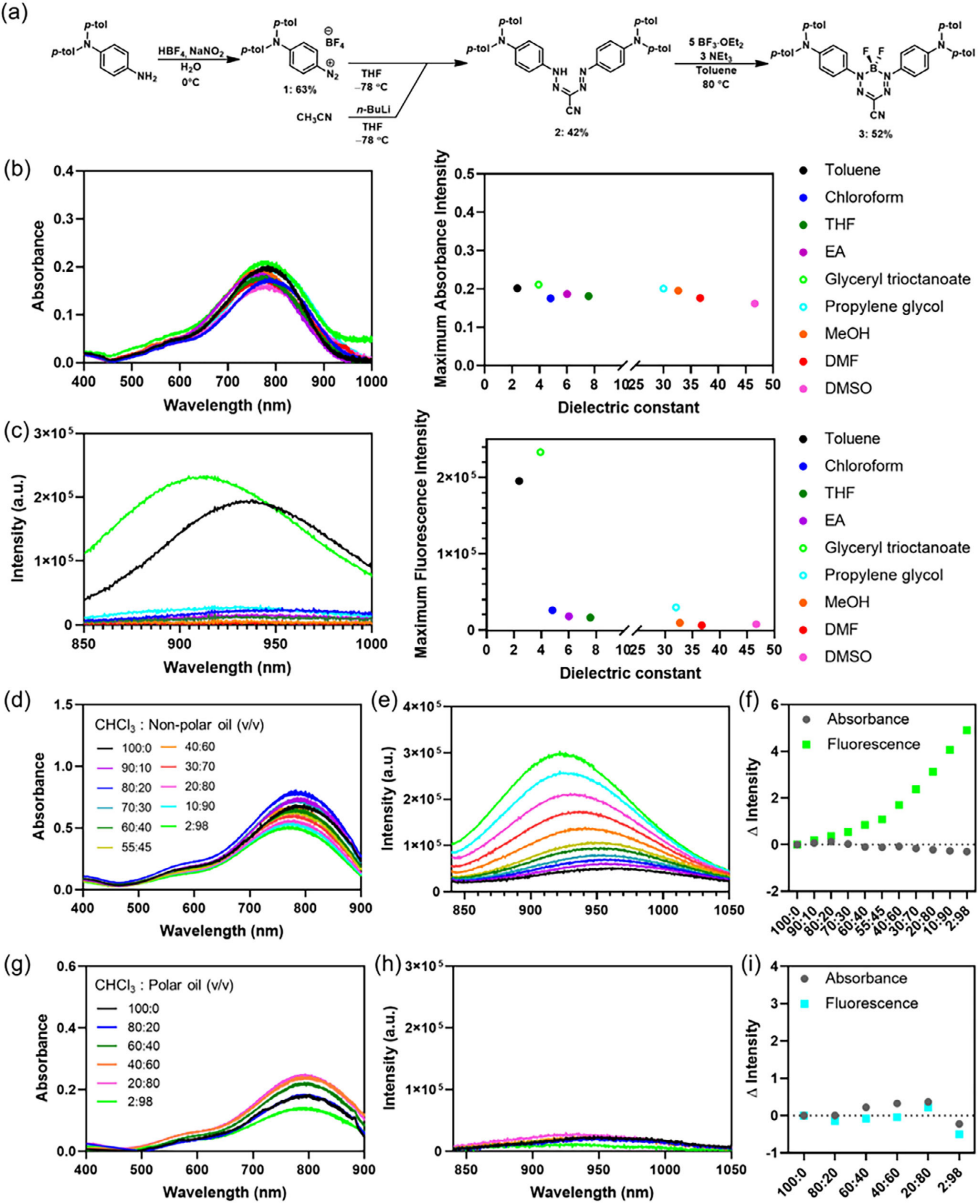
(a) Synthesis scheme of boron difluoride formazanate **3**. (b) UV–vis absorption spectra (left) and absorbance maximum as a function of dielectric constant (right) for **3** (4 µM) in various solvents. (c) Photoluminescence spectra (left) and fluorescence maximum as a function of dielectric constant (right) for **3** (4 µM) in the various solvents. Excitation wavelength: 808 nm. (d–f) Titration of **3** with nonpolar oil (glyceryl trioctanoate) in CHCl_3_. (d) UV–vis absorption spectra, (e) photoluminescence spectra, and (f) relative change in absorbance and fluorescence intensity as a function of oil content. (g–i) Titration of **3** with polar oil in chloroform. (g) UV–vis absorption spectra, (h) photoluminescence spectra, and (i) relative change in absorbance and fluorescence intensity as a function of oil content.

Based on previous structure‐function studies of BF_2_ formazanate dyes [17], we designed compound **3** bearing di‐*p*‐tolylamine groups directly attached to the *N*‐aryl positions to maximize the bathochromic shift while maintaining a relatively high quantum yield. The target BDF dye **3** was synthesized through a three‐step procedure starting from 4‐amino‐*N,N*‐di‐*p*‐tolylaniline (Figure 1a) [28]. Diazonium salt **1** was prepared by diazotization of 4‐amino‐*N,N*‐di‐*p*‐tolylaniline with sodium nitrite in aqueous tetrafluoroboric acid at 0°C (Figure S1). The key formazan intermediate **2,** isolated in 42% yield after purification by column chromatography, was prepared via nucleophilic addition of deprotonated acetonitrile to the diazonium tetrafluoroborate **1** at –78°C by adapting an established protocol [17]. Formation of **2** was confirmed by the appearance of a characteristic NH resonance at 12.97 ppm in the ^1^H NMR spectrum of the purified compound (Figures S2 and S3) and a diagnostic absorbance at 546 nm (ε = 23,200 M^−1^ cm^−1^) in the UV–vis absorption spectrum. Complexation of **2** with BF_3_·OEt_2_ in the presence of NEt_3_ afforded the desired BDF dye **3** in 52% yield after purification by column chromatography. Several spectroscopic features observed for the purified dye confirmed successful synthesis. The ^1^H NMR spectrum showed complete disappearance of the formazan NH signal at 12.97 ppm, indicating successful deprotonation and chelation (Figure S4). The ^11^B NMR spectrum displayed a characteristic triplet at δ = –0.5 ppm (^1^ *J*_BF_ = 31 Hz), indicative of a tetrahedral boron center bound to two fluorine atoms, while the ^19^F NMR spectrum exhibited a quartet at δ = –136.4 ppm (^1^ *J*_FB_ = 28 Hz), confirming the BF_2_ coordination environment (Figure S5). The ^13^C{^1^H} NMR spectrum showed the expected resonances for the formazanate backbone and N‐aryl substituents, with signals at 150.5, 143.6, and 136.4 ppm corresponding to the quaternary carbons (Figure S6). High‐resolution mass spectrometry (ESI^+^) further confirmed the molecular composition, with an [M+H]^+^ ion observed at *m*/z = 688.3166 (calculated: 688.3172). The UV–vis absorption spectrum of **3** in toluene revealed a dramatic bathochromic shift upon boron complexation, with the lowest energy absorption maximum appearing at 785 nm (ε = 47,800 M^−1^ cm^−1^) (Figure S7). Upon photoexcitation, **3** displays near‐infrared emission centered at 898 nm with a quantum yield of 10%, and the emission tail extends beyond 1000 nm, supporting its potential utility as an NIR‐II imaging agent. The large Stokes shift observed between absorption and emission maxima suggests significant structural reorganization in the excited state, consistent with charge‐transfer character in the electronic transition [29]. The significant Stokes shift of 113 nm minimizes self‐absorption and spectral crosstalk, offering key advantages for in vivo fluorescence imaging. Density functional theory (DFT) calculations further elucidated the electronic basis of these optical properties. The highest occupied molecular orbital (HOMO) is distributed across the entire molecular framework, while the lowest unoccupied molecular orbital (LUMO) is localized primarily on the electron‐deficient BF_2_ formazanate core (Figure S8, Table S1). This pronounced donor‐acceptor separation accounts for both the long‐wavelength absorption and the large Stokes shift observed experimentally [18].

### 2.2 Solvent Environment Significantly Influences Optical Properties of Boron Difluoride Formazanate 3

The quantum yield of **3** in toluene was determined to be 10%, a notably high value for organic fluorophores emitting in this spectral range, as most NIR‐II dyes exhibit quantum yields below 4% [30]. However, amine‐substituted BF_2_ formazanates are known to exhibit pronounced sensitivity to their local environment, an important consideration for biological applications where the probe encounters diverse microenvironments. Having established the photophysical properties of **3** in toluene, we next investigated how its optical behavior responds to variation in solvent polarity.

Absorption and fluorescence spectra of **3** were measured in nine solvents spanning a dielectric constant range of ε = 2.4–47 (Figure 1b,c). While the absorption spectra remained largely unchanged across all solvents (Figure 1b, right panel), the fluorescence intensity varied dramatically (Figure 1c, right panel), with bright emission observed exclusively in low‐polarity media. Both toluene (ε = 2.4) and glyceryl trioctanoate (ε ≈ 3.9, estimated from similar triglycerides [31]) yielded fluorescence intensities approximately 10‐fold higher than those observed in polar solvents, and notably higher than even chloroform (CHCl_3_), a conventionally nonpolar solvent. To confirm that polarity, rather than viscosity, governs this behavior, titration experiments were performed using two oils with comparable viscosities but differing polarities (Figure 1d–i). Progressive addition of nonpolar oil (glyceryl trioctanoate) to a CHCl_3_ solution of **3** increased fluorescence intensity by 4.9‐fold at 98% oil content, while the absorption spectra remained unchanged with no baseline elevation or peak broadening, indicating that **3** stayed molecularly dissolved without forming aggregates (Figure 1d–f). In contrast, titration with a polar oil (Propylene glycol) under identical conditions produced negligible fluorescence enhancement (Figure 1g–i). These results clearly indicate that a highly nonpolar environment with a dielectric constant below ∼4 is essential to preserve the NIR‐II quantum yield of **3**.

This differential sensitivity, with polarity‐independent absorption coupled with strongly polarity‐dependent emission, is consistent with prior reports on amine‐substituted BDFs, which have shown that the excited state is significantly more responsive to the solvent environment than the ground state [17, 18, 29, 32]. These observations establish a clear design principle for the biological deployment of **3**. Specifically, shielding **3** from polar environments is essential to maximize its NIR‐II fluorescence quantum yield, while the polarity‐insensitive ground‐state absorption ensures efficient photoacoustic signal generation and photothermal conversion. Guided by this principle, we next sought to engineer a formulation that provides **3** with a nonpolar microenvironment compatible with aqueous biological media.

### 2.3 Design and Synthesis of Boron Difluoride Formazanate 3 Nanoformulation

**FIGURE 2:**
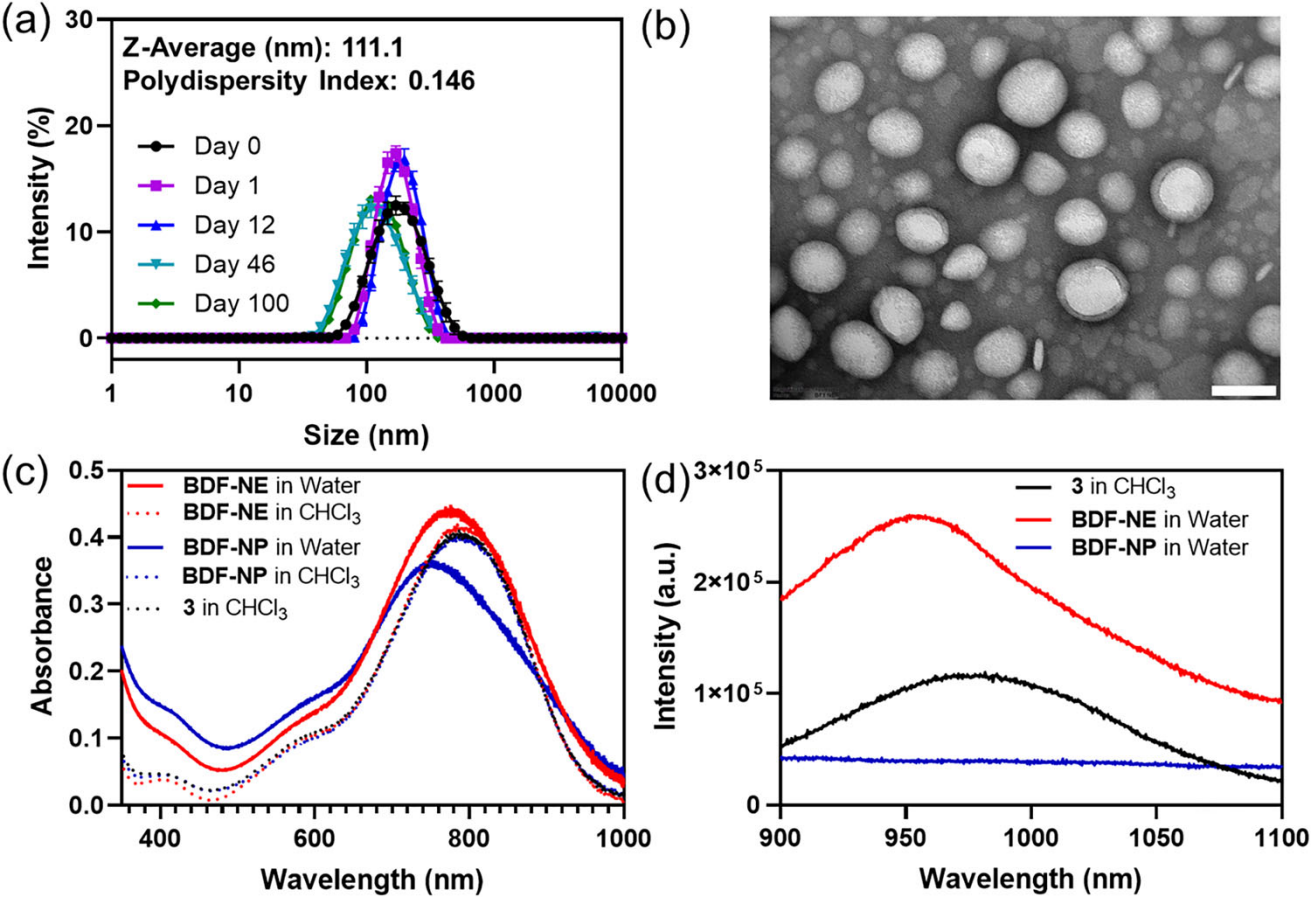
Characterization of BDF‐NE. (a) Size distribution of BDF‐NE measured by dynamic light scattering at day 0, day 1, day 12, day 46, and day 100. (b) Transmission electron microscopy image of BDF‐NE. Scale bar = 100 nm. (c) UV–vis absorption spectra of BDF‐NE in water (red), BDF‐NE in CHCl_3_ (red, dotted), BDF‐NP in water (blue), BDF‐NP in CHCl_3_ (blue, dotted) and **3** in CHCl_3_ (black) at equivalent dye concentration of 18 µM. (d) NIR‐II fluorescence spectra showing enhanced emission from BDF‐NE in water (red) compared to BDF‐NE in CHCl_3_ (black) and BDF‐NP in water (blue) at equivalent dye concentration of 18 µM (λ_ex_ = 808 nm).

To maintain bright NIR‐II emission while preserving robust absorption for PA imaging and PTT in aqueous environments, we reasoned that encapsulating **3** within an oil‐in‐water nanoemulsion could recreate the favorable nonpolar conditions identified in our solvent studies, while simultaneously confining the dye within biocompatible nanoscale droplets that enable stable aqueous dispersibility. Accordingly, we developed a nanoemulsion formulation, denoted **BDF‐NE**, using glyceryl trioctanoate as the nonpolar oil core due to its established pharmaceutical biocompatibility and its ability to provide an optimal microenvironment for both strong absorption and efficient emission (Figure 1). The oil droplets were stabilized by a lipid shell composed of 1,2‐distearoyl‐sn‐glycero‐3‐phosphocholine (DSPC) and 1,2‐distearoyl‐sn‐glycero‐3‐phosphoethanolamine‐*N*‐[methoxy(polyethylene glycol)‐2000] (DSPE‐PEG2000), which conferred robust colloidal stability and PEG‐mediated steric protection to support prolonged in vivo circulation. Dynamic light scattering (DLS) analysis revealed that **BDF‐NE** possessed a hydrodynamic diameter of 111.1 nm with a narrow size distribution (PDI = 0.146), and this size remained stable for at least 3 months at 4°C (Figure 2a), demonstrating excellent colloidal stability. Transmission electron microscopy (TEM) further confirmed a uniform spherical morphology (Figure 2b).

To verify the role of the nonpolar oil core, an oil‐free control formulation was prepared using the same lipid shell components (DSPC and DSPE‐PEG2000) without glyceryl trioctanoate. This oil‐free control formulation, **BDF‐NP**, exhibited a diameter of 130 nm as confirmed by DLS and TEM (Figure S9). To enable concentration‐matched comparison, known volumes of **BDF‐NE** and **BDF‐NP** were disrupted by dissolving in CHCl_3_ to release the encapsulated BDF dye **3**. The absorption spectrum of the disrupted sample was used to determine the exact dye concentration within each nanoformulations, enabling preparation of free BDF dye **3** in CHCl_3_ and BDF nanoformulations in CHCl_3_ and water at identical chromophore concentrations across all samples (Figure 2c, dotted lines). Spectroscopic characterization of **BDF‐NE** validated the underlying design strategy. The UV–vis absorption spectrum of **BDF‐NE** in water closely resembled that of free **3** in CHCl_3_, exhibiting minimal peak broadening or baseline elevation (Figure 2c, red solid line). These features indicate that **3** remains molecularly solubilized within the oil core, thereby preserving its molar extinction coefficient as an essential requirement for efficient light absorption in PA imaging and PTT. However, in contrast to **BDF‐NE**, **BDF‐NP** exhibited a marked decrease in absorption peak intensity compared to free BDF dye **3** in organic solvent, accompanied by a 40 nm hypsochromic shift, indicative of dye aggregation in the absence of the nonpolar oil core (Figure 2c, blue solid line). Fluorescence measurements further highlighted the photophysical advantages of the nanoemulsion platform. At the matched dye concentration, **BDF‐NE** in water displayed substantially enhanced NIR‐II emission relative to free **3** in CHCl_3_, accompanied by a slight blue shift (Figure 2d, red solid line). This spectral blue shift is consistent with the behavior observed in solvent titration experiments when **3** was transferred from CHCl_3_ to the more nonpolar glyceryl trioctanoate environment (Figure 1e), confirming that the nanoemulsion core provides the intended low‐polarity microenvironment. The elevated baseline observed in the fluorescence spectrum is attributed to light scattering from the nanoscale droplets. Despite extensive optimization, complete elimination of scattering under the measurement conditions was not achievable. To further verify fluorescence enhancement while minimizing scattering contribution, additional measurements were performed in the NIR‐II region (>1000 nm), where scattering of both excitation and emission light is reduced. Although only a portion of the emission profile could be captured within this spectral window, the recorded spectra clearly showed enhanced and slightly blue‐shifted fluorescence for **BDF‐NE** relative to free **3** in CHCl_3_ (Figure S10). In contrast, **BDF‐NP** exhibited complete fluorescence quenching under identical measurement conditions (Figure 2d, blue solid line), confirming that the nonpolar glyceryl trioctanoate core is essential for bright NIR‐II emission. These results together confirm that the nanoemulsion formulation successfully provides an intended nonpolar microenvironment that preserves the bright emission of **3** in aqueous media and retains an emission tail extending into the NIR‐II region, supporting its suitability for deep‐tissue imaging applications.

### 2.4 Photothermal Efficacy of BDF‐NE

**FIGURE 3:**
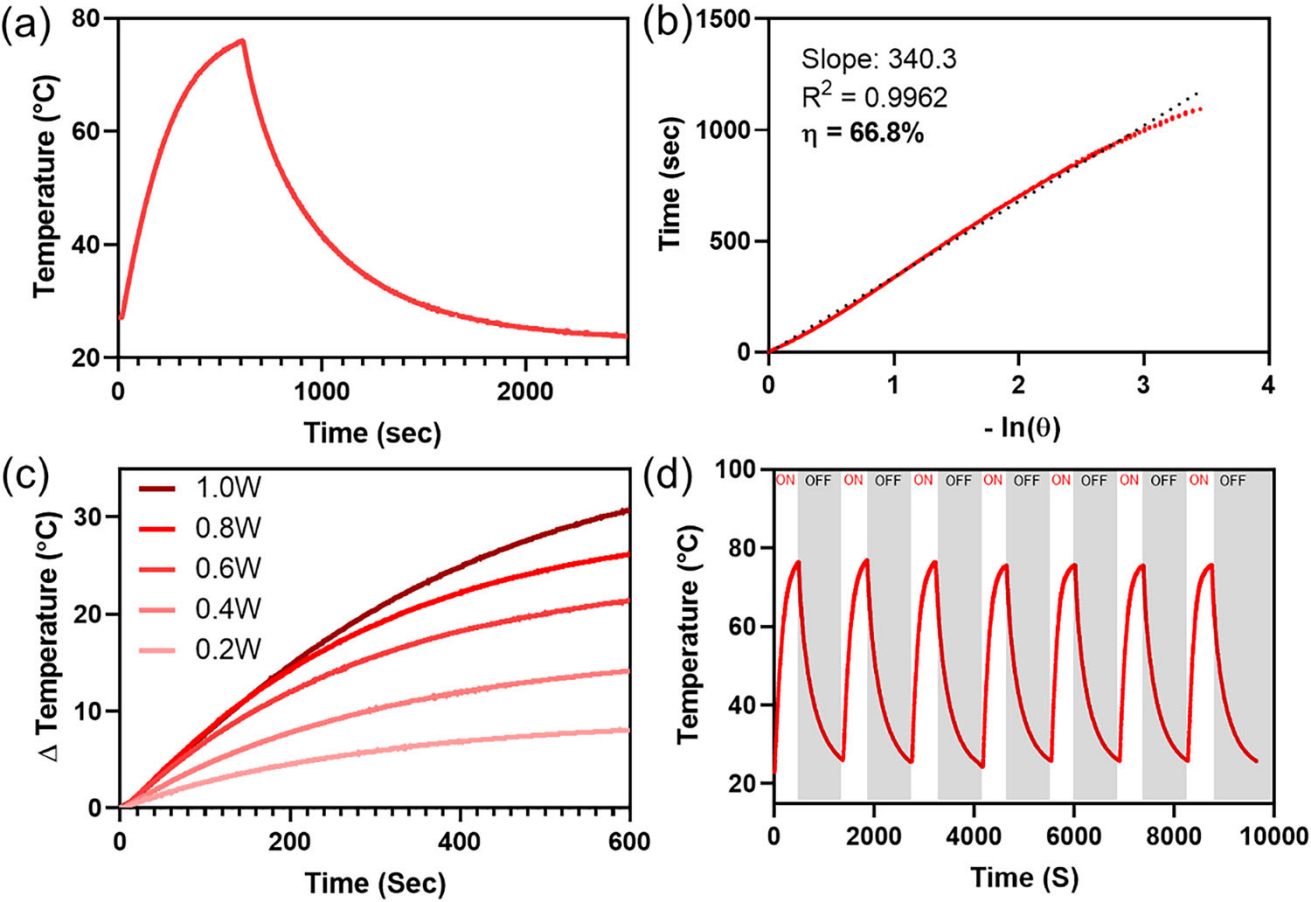
Photothermal properties of BDF‐NE. (a) Temperature elevation profile of BDF‐NE upon 825 nm laser irradiation (1.0 W cm^−2^) and subsequent cooling after laser cessation. (b) Linear fitting of time constant versus negative natural logarithm of temperature driving force for photothermal conversion efficiency calculation, yielding η = 66.8% (*R* ^2^ = 0.9962). (c) Power density‐dependent temperature rise of BDF‐NE under 825 nm laser irradiation at varying power densities (0.2–1.0 W cm^−2^). (d) Temperature cycling profile of BDF‐NE over seven ON/OFF heating‐cooling cycles under 825 nm laser irradiation, demonstrating excellent photothermal stability.

Having established that **BDF‐NE** preserves the strong absorptivity of **3** while markedly enhancing its NIR‐II fluorescence, we next evaluated whether **BDF‐NE** could effectively translate optical absorption into robust photothermal performance. The temperature of an aqueous **BDF‐NE** dispersion was monitored under continuous 825 nm laser irradiation at 1.0 W cm^−^^2^. Rapid thermal elevation was observed, with the solution temperature rising to approximately 76°C within 300 s (Figure 3a), confirming efficient light‐to‐heat conversion. The photothermal conversion efficiency (PCE) was then determined through analyzing the heating‐cooling profiles. Linear fitting of the time constant against the negative natural logarithm of the normalized temperature driving force yielded an excellent correlation (*R* ^2^ = 0.9962), corresponding to a PCE of 66.8% (Figure 3b). To our knowledge, this value ranks among the highest reported for BDF‐based photothermal agents (Table S2). A key consideration is whether preserving the fluorescence quantum yield of **3** by encapsulation within the oil core compromises photothermal efficiency. To directly address this, we compared the photothermal heating profiles of **BDF‐NE** and **BDF‐NP**, under optical density‐matched conditions at 825 nm. Both formulations exhibited virtually identical heating profiles (Figure S11), demonstrating that the glyceryl trioctanoate oil core does not affect photothermal conversion efficiency. These results confirm that the nanoemulsion formulation maintains efficient photothermal conversion. We next examined the dependence of photothermal heating on laser power density and assessed the photostability of **BDF‐NE** under repeated irradiation. Temperature increase scaled approximately linearly with incident power density over the range of 0.2 to 1.0 W cm^−^^2^ (Figure 3c), which confirms that heating generation raised directly from optical absorption rather than secondary photochemical processes. This predictable scaling enables precise control of thermal dose during treatment. Moreover, under seven consecutive heating‐cooling cycles at 1.0 W cm^−^^2^, both the peak temperature and heating kinetics remained essentially unchanged (Figure 3d), indicating excellent photothermal stability without detectable photobleaching or thermal degradation.

Collectively, these results demonstrate that the nanoemulsion formulation satisfies the dual requirements for effective BDF‐based theranostics. The nonpolar oil core preserves the bright NIR‐II fluorescence of **3**, while the intrinsic polarity‐insensitive absorption of BDF dyes ensures strong optical absorption and efficient photothermal conversion. This dual functionality positions **BDF‐NE** as a versatile theranostic platform capable of simultaneously delivering robust PA contrast and bright NIR‐II fluorescence to guide PTT.

### 2.5 Photoacoustic Imaging Enabled by BDF‐NE

**FIGURE 4:**
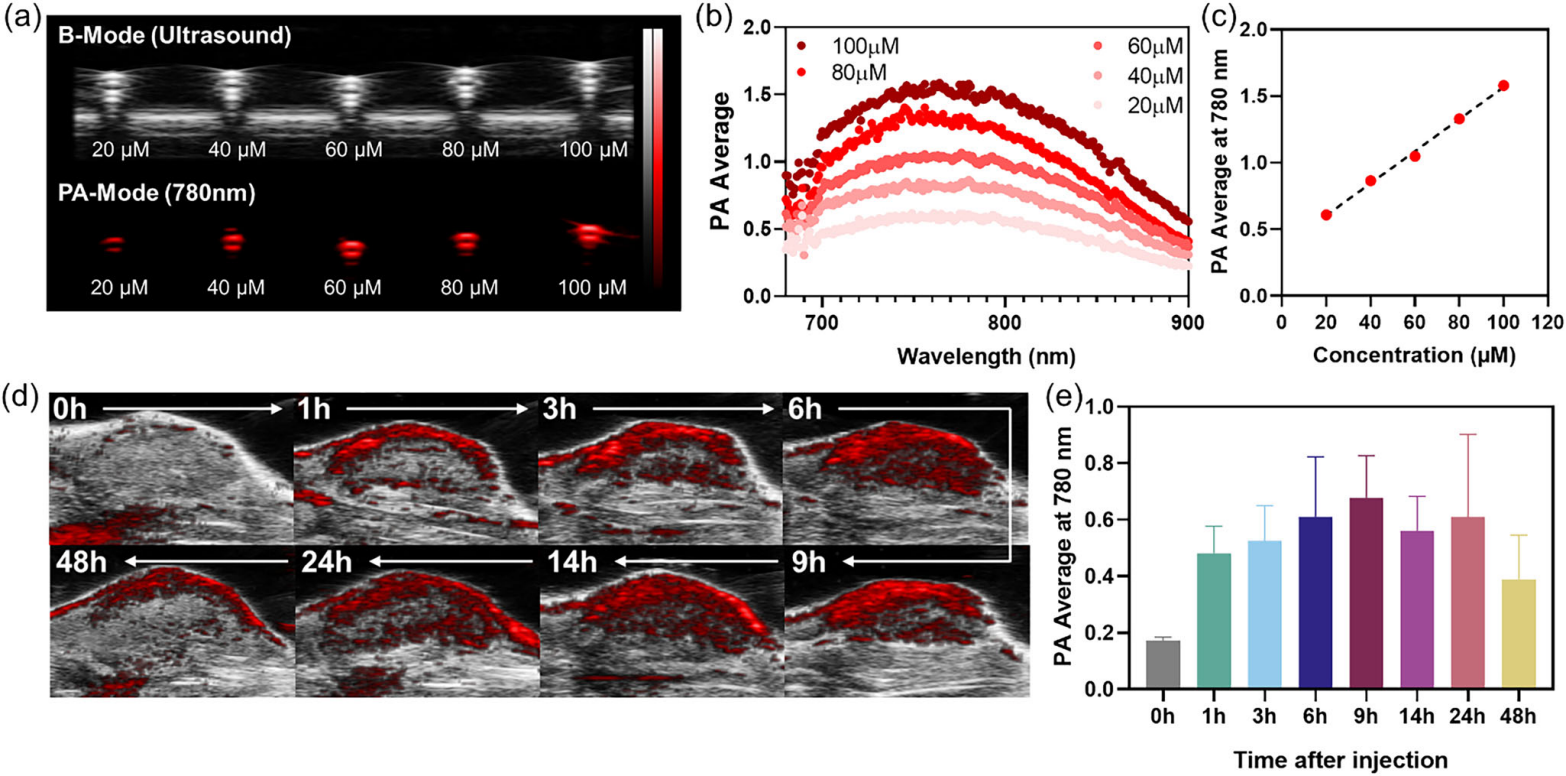
Photoacoustic (PA) imaging performance of BDF‐NE in phantom and in vivo. (a) Ultrasound (top) and PA image at 780 nm (bottom) of BDF‐NE solutions at concentrations ranging from 20 to 100 µM in thin tube phantoms. (b) Wavelength‐dependent PA signal intensity of BDF‐NE at various concentrations, showing maximum signal near 780 nm. (c) Linear correlation between PA signal intensity at 780 nm and BDF‐NE concentration (*R* ^2^ > 0.99). (d) Time‐resolved PA imaging of KB tumor‐bearing mice following intravenous injection of BDF‐NE, with grayscale ultrasound overlaid with PA signal (red). (e) Quantification of PA signal intensity at 780 nm in tumor regions at different time points post‐injection, demonstrating peak accumulation at 6–9 h.

As described, the strong NIR absorption of **BDF‐NE** at 785 nm, combined with its high PCE, suggests its excellent potential for PA imaging. This spectral window is particularly advantageous for in vivo PA imaging, as hemoglobin absorption diminishes substantially above 750 nm, while water absorption remains negligible below 900 nm, thereby minimizing interference from endogenous chromophores. We first evaluated the PA response of **BDF‐NE** using phantom studies. Solutions with concentrations ranging from 20 to 100 µM were imaged with 780 nm excitation (Figure 4a). Spatial reference was provided by ultrasound, while PA imaging revealed clear concentration‐dependent variations in signal intensity. Spectral analysis across the 680–900 nm range confirmed that the PA signal peaked near 780 nm for all concentrations (Figure 4b), in consistent with the absorption maximum observed in UV‐vis spectroscopy. A highly linear relationship (*R* ^2^> 0.99) between PA signal intensity and concentration was observed (Figure 4c), suggesting that **BDF‐NE** holds promise as a quantitative PA contrast agent for noninvasive mapping of local agent accumulation in tissue.

To assess whether these characteristics are available in vivo, we performed time‐resolved PA imaging in KB tumor‐bearing mice following intravenous administration of **BDF‐NE**. Ultrasound provided anatomical localization of the subcutaneous tumor, while PA signal at 780 nm tracked the biodistribution of **BDF‐NE** over time (Figure 4d and S12). At the baseline of pre‐injection (0 h), minimal PA signal was detected in the tumor region. Signal intensity increased progressively over the first 6 h, reaching a maximum between 6 to 9 h post‐injection. Quantitative analysis of tumor PA intensity revealed a ∼3‐fold enhancement at peak accumulation relative to baseline (Figure 4e), indicating substantial retention of **BDF‐NE** in the tumor. After 24 h, the PA signal gradually declined, consistent with the clearance of **BDF‐NE** from the tumor site. These results demonstrated that **BDF‐NE** functions as an effective PA contrast agent in both solution and in vivo tumor models. Its ability to generate strong, concentration‐dependent PA signals to monitor dynamics of tumor accumulation in vivo supports its use as a contrast agent for PA image‐guided PTT.

### 2.6 In Vivo Photothermal Therapy With BDF‐NE

**FIGURE 5:**
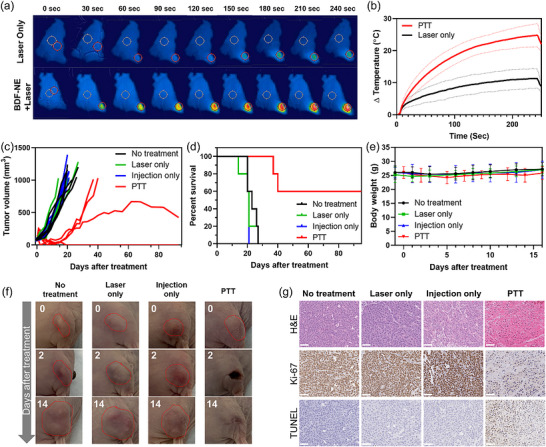
In vivo photothermal therapeutic efficacy of BDF‐NE in KB tumor‐bearing mice. (a) Infrared thermal images of tumor‐bearing mice during 825 nm laser irradiation (1.0 W cm^−2^, 4 min). Top row: laser only control; bottom row: BDF‐NE + laser (PTT group). (b) Temperature elevation profiles at tumor sites monitored by thermocouple for PTT group (red) and laser only control (black). Dotted lines represent standard deviation; solid lines represent mean values. (c) Tumor growth curves monitored over 90 days for all treatment groups (n = 5 per group). (d) Kaplan‐Meier survival curves for mice in different treatment groups. (e) Body weight changes during the 16‐day observation period, showing no significant differences among groups. (f) Representative photographs of tumor‐bearing mice at days 0, 2, and 14 post‐treatment for four groups: no treatment (NT), laser only, injection only (BDF‐NE without laser), and PTT (BDF‐NE + laser). Red circles indicate tumor locations. (g) Histological analysis of tumor tissues harvested 2 days post‐treatment. Top row: H&E staining; middle row: Ki‐67 staining; bottom row: TUNEL staining. Scale bars: 50 µm.

The combination of high PCE (66.8%) and favorable tumor accumulation kinetics (peak uptake at 6–9 h post‐injection) established **BDF‐NE** as a promising candidate for in vivo PTT. We first assessed whether laser irradiation could elevate intratumoral temperature to therapeutically relevant levels in mice treated with **BDF‐NE**. KB tumor‐bearing mice were divided into two groups, with only one group receiving intravenous **BDF‐NE**. At 6 h post‐injection, tumors of both groups were exposed to identical 825 nm laser irradiation (1.0 W cm^−2^), while tumor temperature was monitored using both an infrared thermal camera (surface) and an implanted thermocouple (intratumoral core). In **BDF‐NE**‐treated mice, tumor temperature increased rapidly during the initial 2 min of irradiation, with both surface and intratumoral temperatures exceeding 50°C (Figure 5a,b). Temperatures continued to rise more gradually over the next 2 min, reaching a peak intratumoral temperature of 54°C at 4 min, at which point laser irradiation was terminated. In contrast, tumors subjected to laser alone showed only modest heating, with final temperatures remaining below 40°C. These results confirmed that **BDF‐NE** enables efficient, localized tumor heating sufficient to induce PTT‐mediated thermal damage, and that temperature elevation is attributable to **BDF‐NE** rather than intrinsic tissue absorption. These results established the PTT treatment protocol, 825 nm laser irradiation at 1.0 W cm^−2^ for 4 min.

To assess therapeutic efficacy, KB tumor‐bearing mice were randomized into four groups (*n* = 5 per group): (I) no treatment (NT), (II) laser only, (III) **BDF‐NE** only (without laser), and (IV) PTT (**BDF‐NE** + laser). This groups design allowed independent assessment of the biological effects of laser exposure and nanoemulsion administration. Tumors were irradiated at 6 h post‐injection, and tumor volumes were monitored over 90 days (Figures 5c, S13‒16). In all three control groups, tumors showed rapid progression, and all mice reached endpoint criteria within 27 days. In contrast, the PTT group exhibited dramatic therapeutic benefit. Two animals achieved complete tumor ablation with no recurrence throughout the 93‐day observation period, while the remaining mice showed substantially delayed tumor growth. Survival analysis reinforced significantly prolonged survival in the PTT group compared to all control groups (Figure 5d). Body weight remained stable across all groups, and histological examination of major organs revealed no signs of systemic toxicity (Figures 5e, S17). Representative photographs (Figure 5f) illustrated the treatment responses, with black eschar and localized edema visible at PTT treatment sites by day 2, consistent with acute thermal damage. These lesions gradually resolved and appeared nearly normal by day 14, while tumors in control groups continued to expand.

To elucidate the cellular mechanisms underlying tumor ablation, tumor tissues were collected 2 days post‐treatment for histological analysis. H&E staining of PTT‐treated tumors revealed extensive necrosis, characterized by cytoplasmic eosinophilia and loss of tissue architecture (Figure 5g, top). In contrast, tumors in the NT, laser only, and **BDF‐NE** only groups displayed preserved morphology with minimal impact. Ki‐67 staining demonstrated dense nuclear staining in control tumors, while PTT‐treated tumors showed markedly reduced Ki‐67 expression (Figure 5g, middle row), indicating suppression of proliferative activity. TUNEL staining revealed substantial apoptotic DNA fragmentation in PTT‐treated tumors but negligible in control tissues (Figure 5g, bottom), demonstrating apoptosis as an additional mechanism of tumor cell death. Collectively, these data establish that **BDF‐NE**‐mediated PTT enables potent tumor ablation through combined photothermal‐induced necrosis and apoptosis. The therapy produced complete tumor eradication in a majority of treated animals, significantly prolonged survival, and induced no detectable systemic toxicity. Histological analyses confirmed that therapeutic efficacy derives from direct thermal damage to tumor tissue with minimal off‐target impact. These results position **BDF‐NE** as an effective theranostic nanoplatform that integrates multimodal imaging capability with highly effective PTT.

### 2.7 In Vivo NIR‐II Fluorescence Imaging With BDF‐NE

**FIGURE 6:**
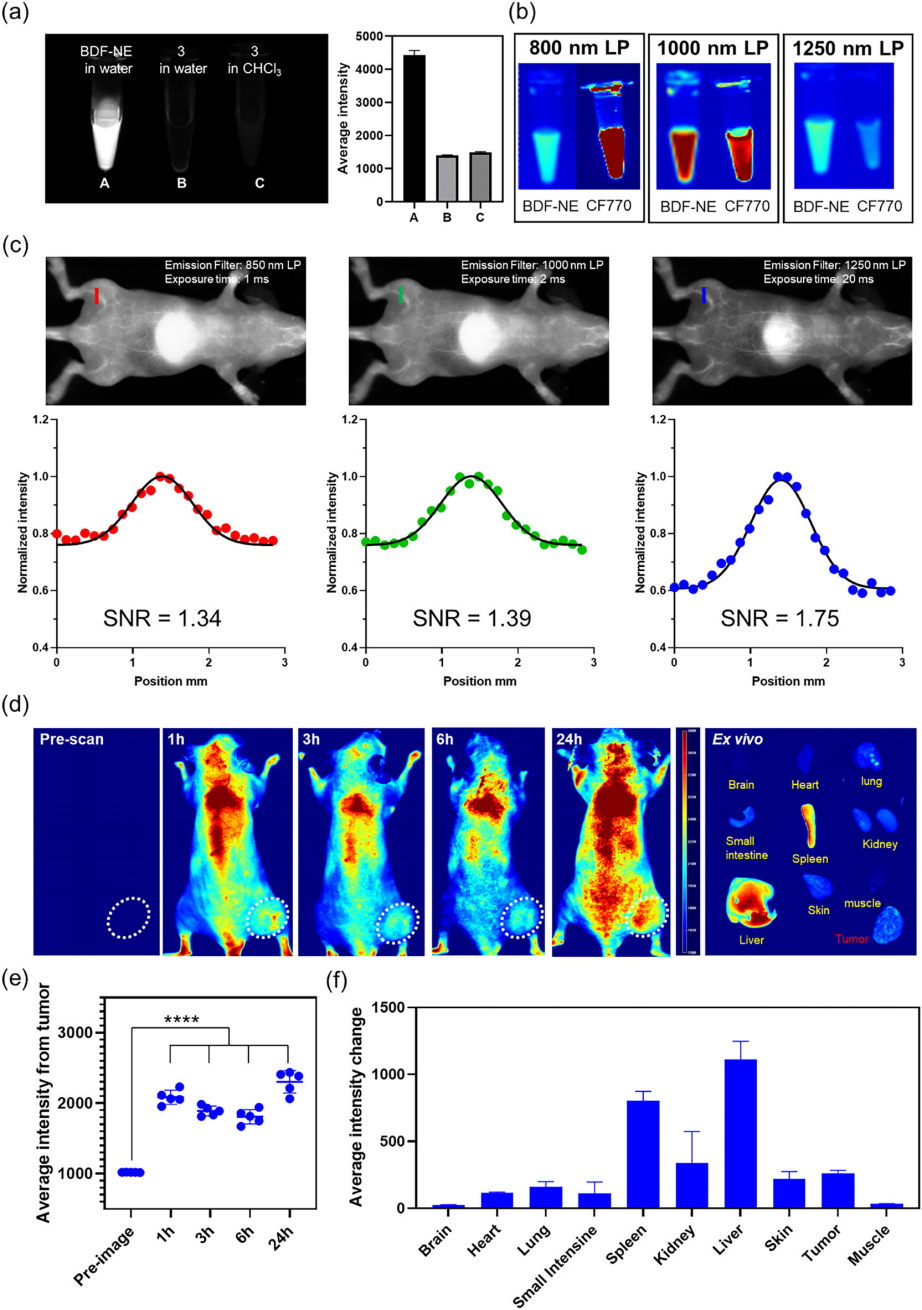
NIR‐II fluorescence imaging performance of BDF‐NE. (a) NIR‐II fluorescence images (left) and quantified average intensities from ROI (right) of (A) BDF‐NE in water, (B) **3** in water, and (C) **3** in CHCl_3_ at equivalent chromophore concentrations. Images were acquired using a small animal imaging system with 785 nm excitation and 800 nm long‐pass filter. (b) NIR‐II fluorescence images of BDF‐NE (50 µM) and CF770 (50 µM) solutions captured through 800 nm, 1000 nm, and 1250 nm longpass (LP) filters. (c) High‐resolution NIR‐II fluorescence imaging of hindlimb vasculature in mice 5 min post‐injection of BDF‐NE, acquired through 850, 1000, and 1250 nm LP. Bottom panels show normalized fluorescence intensity profiles across vessels (marked by red lines). (d) Time‐resolved whole‐body NIR‐II fluorescence imaging of KB tumor‐bearing mice following intravenous injection of BDF‐NE (1250 nm LP filter). White dashed circles indicate tumor region. Right panel shows ex vivo fluorescence imaging of dissected organs at 24 h post‐injection. (e) Quantification of tumor fluorescence intensity over time. Tumor fluorescence intensity at all time points was significantly higher than pre‐injection baseline (***p < 0.0001, one‐way ANOVA with Dunnett's test, *n* = 5) (f) Quantitative analysis of ex vivo organ fluorescence at 24 h post‐injection.

In addition to its photothermal therapeutic function, BDF‐NE offers a complementary imaging modality that directly benefits from its nanoemulsion formulation. As demonstrated earlier, the nonpolar oil‐core environment significantly enhances the fluorescence output of BDF **3**, producing emission that extends well into the NIR‐II region. We therefore investigated whether this enhancement translates into improved in vivo NIR‐II fluorescence imaging performance. We first compared the NIR‐II fluorescence intensity of BDF‐NE with control samples using a small animal imaging system. At equivalent chromophore concentrations, BDF‐NE in water exhibited dramatically stronger fluorescence compared to both free **3** in water (0.5% THF) and free **3** in CHCl_3_ (Figure 5a). Free **3** in water showed minimal fluorescence due to aggregation and polarity‐induced quenching, while free **3** in CHCl_3_ displayed moderate emission consistent with our solvent studies. In contrast, BDF‐NE produced approximately 3‐fold higher fluorescence intensity than **3** in CHCl_3_, demonstrating that the nanoemulsion formulation successfully provides the nonpolar microenvironment necessary to maximize NIR‐II emission in aqueous media. Hyperspectral emission profiling revealed that **BDF‐NE** in aqueous medium exhibited a peak emission near 980 nm with an extended tail beyond 1250 nm (Figure S18). This broad emission range (900–1300 nm) positions **BDF‐NE** favorably for NIR‐II imaging, where tissue autofluorescence, hemoglobin absorption, and photon scattering are minimized. To benchmark this long‐wavelength performance, **BDF‐NE** was compared with CF770 (Biotium) under identical excitation and detection conditions using 800, 1000, and 1250 nm long‐pass (LP) filters (Figure 6b). CF770 is a widely used NIR fluorophore excited in the NIR‐I region (∼780 nm) with a broad emission extending into the NIR‐II window, and was selected as a reference owing to its closely matching excitation profile with **BDF‐NE**. At 800 nm LP, CF770 displayed stronger signal, consistent with its small Stokes shift and emission maximum close to the excitation wavelength. At 1000 nm LP, both probes produced comparable emission. At 1250 nm LP, however, **BDF‐NE** retained a clear detectable signal, whereas CF770 approaches background levels. This crossover behavior reflects the extended NIR‐II emission tail and larger Stokes shift of **BDF‐NE**, enabling efficient photon detection ≥1200 nm where reduced scattering and autofluorescence and improved deep‐tissue imaging, while simultaneously minimizing contribution from excitation light and shorter‐wavelength emissions. To further characterize the NIR‐II brightness of **BDF‐NE**, the absolute NIR‐II fluorescence quantum yield was measured using an integrating sphere under 808 nm excitation with a 1000 nm long‐pass filter, yielding a value of 2.9% (1000 nm‒1800 nm, see Supporting Information for measurement details). We next assessed spatial resolution and tissue penetration performance in vivo by imaging the hindlimb vasculature of mice at 5 min post‐injection. Blood vessels were clearly visualized using 850, 1000, and 1250 nm LP filters (Figure 6c). Cross‐sectional intensity profiles demonstrated consistent vascular delineation across all wavelength windows, confirming that **BDF‐NE** generates sufficient photon flux even in the deeper NIR‐II region. The signal‐to‐noise ratio (SNR) of 1.39 at 1000 nm LP compares favorably with ICG, the only clinically approved NIR fluorophore with a comparable excitation profile, which has been reported to yield SNR values below 1.26 under 808 nm excitation with a 1000 nm long‐pass filter [33]. Notably, imaging at 1250 nm LP yielded the highest SNR of 1.75, consistent with reduced scattering and autofluorescence at longer wavelengths.

To probe tumor‐targeted imaging performance, we preformed time‐resolved whole‐body NIR‐II imaging in KB tumor‐bearing mice following intravenous **BDF‐NE** administration(Figure 6d). At 1 h post‐injection, fluorescence traced major blood vessels, indicating effective systemic circulation. Distinct tumor‐associated fluorescence emerged by 3 h, driven by the enhanced permeability and retention (EPR) effect that promotes nanoemulsion accumulation in tumors with leaky vasculature and poor lymphatic drainage. Tumor fluorescence peaked at 24 h, although background signals increased due to redistribution of **BDF‐NE** through hepatobiliary clearance and uptake into the reticuloendothelial system (RES). *Ex vivo* organs’ imaging at 24 h (Figure 6f) confirmed highest fluorescence in liver and spleen, characteristic of RES‐mediated nanoparticles clearance. Tumor tissue exhibited substantial fluorescence signal, comparable to kidney and notably higher than heart, lung, brain, or muscle. Importantly, this biodistribution pattern closely matches the PA imaging results, confirming robust tumor accumulation with relatively low signal in normal tissues during the early post‐injection period. The strong tumor contrast at 6 h, coupled with low background in healthy organs, suggests this time point as optimal for imaging and therapeutic intervention, supporting the 6 h treatment window used in our PTT studies.

Overall, these NIR‐II fluorescence imaging results, together with the PA imaging and PTT efficacy demonstrated above, establish **BDF‐NE** as a comprehensive and effective theranostic platform. The oil‐in‐water nanoemulsion formulation not only promotes aqueous stability and nanoassembly enhanced PTT but also enhances the intrinsic fluorescence of **3**, unlocking NIR‐II imaging capability typically inaccessible to conventional nanoparticle system.

## 3 Conclusion

In this study, we have synthesized a new BDF dye **3** via a concise three‐step route and successfully formulated it into an oil‐in‐water nanoemulsion (**BDF‐NE**). Systematic solvent studies revealed that the fluorescence of **3** is highly polarity‐sensitive, with bright emission only in environments with dielectric constants below ∼4, while absorption remains largely unaffected. Guided by this insight, **BDF‐NE** was designed with a nonpolar glyceryl trioctanoate core that enable bright NIR‐II fluorescence of **3** in aqueous media without compromising absorptivity. Notably, when **3** was formulated into oil‐free lipid nanoparticles under otherwise identical conditions, its fluorescence was completely quenched, whereas **BDF‐NE** achieved a quantum yield of 2.9% beyond 1000 nm, directly confirming that the nonpolar oil core is essential for preserving the emissive properties of **3**. **BDF‐NE** exhibits a PCE of 66.8%, among the highest reported for BDF‐based systems, demonstrating that bright NIR‐II fluorescence and efficient photothermal activity can coexist within this single platform. These photophysical properties enable dual‐modal imaging, producing linear, concentration‐dependent PA signals near 780 nm and bright NIR‐II fluorescence beyond 1250 nm, allowing high‐contrast vascular imaging and precise visualization of tumor accumulation with strong concordance between PA and fluorescence signals. Leveraging these imaging capabilities, a single dose **BDF‐NE** mediated PTT induced rapid intratumoral heating above 50°C, triggering extensive necrosis and apoptosis. Most treated animals exhibited significant tumor suppression, and a subset achieved complete regression with long‐term, recurrence‐free survival over a 90‐day, all without detectable systemic toxicity. To our knowledge, **BDF‐NE** is the first nanoemulsion formulation of BDF dyes, demonstrating that nonpolar microenvironment engineering via nanoemulsion can simultaneously maximize fluorescence, preserve photothermal efficiency, and ensure stable aqueous dispersibility. This strategy provides a generalizable approach for translating hydrophobic, polarity‐sensitive NIR chromophores into high‐performance dual‐modal theranostic agents.

## Author Contributions

N.K. conceptualized the study, designed and performed the experiments, analyzed the data, and wrote the manuscript. F.L.B. designed and synthesized the BDF compound. T.H. set up the NIR fluorescence measurement system. S.C. assisted with material characterization. D.Z., B.L., U.I., M.W., and A.V. performed the in vivo NIR‐II imaging experiments. K.H., J.W. and B.R. provided access to the NIR‐II spectrometer and assisted with NIR‐II emission characterization. J.C. contributed to nanoparticle formulation, project guidance, and manuscript revision. J.B.G. and G.Z. supervised the project.

## Conflicts of Interest

The authors declare no conflicts of interest.

## Supporting information




**Supporting File**: The authors have cited additional references within the Supporting Information [19, 20, 21, 22, 23, 24, 25, 26, 28, 34, 35, 36, 37].

## Data Availability

The data that support the findings of this study are available from the corresponding author upon reasonable request.
