# Supplementary material for: Engineering Nanoemulsions to Maximize NIR‐II Fluorescence and Preserve Photothermal Performance of a Novel Boron Difluoride Formazanate Dye
Source: Angew Chem Int Ed Engl. 2026 May 4;65(25):e4378015. doi: 10.1002/anie.4378015 (PMC13266931; doi:10.1002/anie.4378015)
Supplement: Supplementary file 1 — Supporting File: The authors have cited additional references within the Supporting Information [19, 20, 21, 22, 23, 24, 25, 26, 28, 34, 35, 36, 37]. [file ANIE-65-e4378015-s001.pdf]

Supporting Information

Engineering Nanoemulsions to Maximize NIR-II Fluorescence and Preserve Photothermal Performance of a Novel Boron Difluoride Formazanate Dye

Nahyun Kwon,[a] Francis L. Buguis,[b] Theo Husby,[a,c] Suhjung Chun,[b] Dongling Zhang,[d] Jiaze Wu,[e] Benjamin Rehl,[f] Binbing Ling,[d] Umar Iqbal,[d] Melissa Washington,[d] Angie Verner,[d] Kai Huang,[e] Juan Chen,[a] Joe B. Gilroy,*[b] Gang Zheng*[a,c]

[a] Dr. N. Kwon, Dr. J. Chen, Prof. Dr. G. Zheng
Princess Margaret Cancer Centre
University Health Network
101 College Street, Toronto, Ontario, M5G 1L7 Canada
E-mail: gang.zheng@uhn.ca

[b] Dr. F. L. Buguis, S. Chun, Prof. Dr. J. B. Gilroy
Department of Chemistry
The University of Western Ontario
1151 Richmond Street North, London, Ontario, N6A 5B7 Canada
E-mail: joe.gilroy@uwo.ca

[c] T. Husby, Prof. Dr. G. Zheng
Department of Medical Biophysics
University of Toronto
Toronto, Ontario, Canada

[d] D. Zhang, B. Ling, U. Iqbal, M. Washington, A. Verner
Human Health Therapeutics Research Center
National Research Council Canada
Ottawa, Ontario, K1A 0R6, Canada

[e] J. Wu, Prof. Dr. K. Huang
Department of Materials Science and Engineering
University of Toronto
Toronto, Ontario, M5S 3E4, Canada

[f] Dr. B. Rehl
Department of Electrical and Computer Engineering
University of Toronto
10 King's College Road, Toronto, Ontario, M5S 3G4 Canada

Materials and Methods

Synthesis and characterization of 3 Reagents were purchased from Sigma-Aldrich, Alfa Aesar, or Oakwood Products Inc. and used as received unless otherwise specified. The synthesis of 4-nitro-*N,N*-di-*p*-tolylaniline have been reported previously.[1] Solvents were purchased from Caledon Laboratories, dried using an Innovative Technologies Inc. solvent purification system, collected under vacuum, and stored under N_2 over 4 Å molecular sieves. Reactions were performed under an N_2 atmosphere using standard Schlenk techniques unless otherwise stated. NMR spectra were recorded on 400 MHz (^{1}H: 399.8 MHz, ^{11}B: 128.3 MHz, ^{19}F: 376.1 MHz) or 600 MHz (^{1}H: 599.5 MHz, ^{13}C{^{1}H}: 150.8 MHz) Varian INOVA spectrometers. ^{1}H NMR spectra were referenced to residual tetramethylsilane (δ: 0.00) using the residual $CHCl_3$ (δ: 7.26) or DMSO-d_5 (δ: 2.50) solvent signals and ^{13}C{^{1}H} NMR spectra were referenced to tetramethylsilane (δ:0.00) using the $CDCl_3$ (δ: 77.0) solvent signal. ^{11}B NMR spectra were referenced to $BF_3{\cdot}OEt_2$ (δ: 0.0), and ^{19}F NMR spectra were referenced to $CFCl_3$ (δ: 0.0) Mass spectrometry data were recorded in positive-ion mode using a high–resolution Thermo Scientific DFS (Double Focusing Sector) mass spectrometer using electron impact or Bruker microTOF II spectrometer using electrospray ionization (ESI). Solution UV-vis absorption spectra in toluene were recorded using a Cary 5000 UV-vis-NIR spectrophotometer. Molar extinction coefficients were determined from the slope of a plot of absorbance against concentration using four solutions with different known concentrations. Photoluminescence measurements were conducted in toluene (5 μM) using an Acton 2300i spectrograph equipped with an iDus BRDD CCD camera. A 532 nm COHERENT laser (model 1037860, VERDI 5W) attached to a cooler (model: T255p) was utilized to excite the boron difluoride formazanate dye **3**.

Synthesis of 4-(di-*p*-tolylamino)benzene diazonium tetrafluoroborate **1**

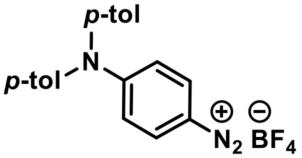

In air, a solution of 4-amino-*N,N*-di-*p*-tolylaniline (8.27 g, 28.7 mmol) in water (100 mL) was cooled to 0 °C for 10 min before tetrafluoroboric acid (8.81 mL, 12.6 g, 143 mmol) was added. The suspension was stirred at 0 °C for 20 min and a cold solution of sodium nitrite (2.18 g, 31.6 mmol) in water (20 mL) was added dropwise and the solution was stirred for 3 h. The brown product was collected by vacuum filtration and washed with excess water. Yield: 7.71 g, 69%. ^{1}H NMR (600 MHz, DMSO-d_6): δ 8.24 (d, $^{3}J_{HH}$ = 9 Hz, 2H, aryl C*H*), 7.36–7.32 (m, 8H, aryl C*H*), 6.79 (d, $^{3}J_{HH}$ = 9 Hz, 2H, aryl C*H*), 2.34 (s, 6H, C*H*).

Synthesis of Formazan **2**

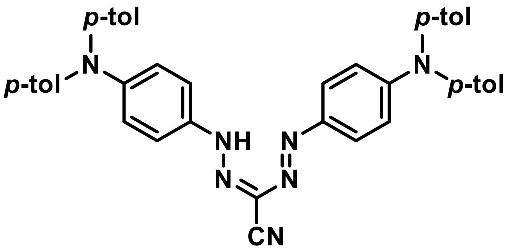

In air, a solution of CH_3CN (2.08 mL, 39.8 mmol) in dry THF (100 mL) was cooled at −78 °C for 30 min. Then, 2.5 M *n*-BuLi (9.16 mL, 22.9 mmol) was added dropwise to the solution over a period of 15 min. A suspension of diazonium tetrafluoroborate **1** (7.71 g, 19.9 mmol) in dry THF (200 mL) was cooled separately to −78 °C and stirred for 5 min before it was added dropwise to the first solution containing CH_3CN. The resulting dark red solution was stirred for

6 h at –78 °C before it was warmed to room temperature over 12 h. The reaction was quenched with ethanol (5 mL), and the solvent was removed under vacuum. The solids were purified using column chromatography (CH_2Cl_2, neutral alumina, R_f = 0.88). Then, the solvent was removed *in vacuo*, and the resulting solids were further purified by trituration with ice cold methanol. Yield = 2.65 g, 42%. ^{1}H NMR (400 MHz, $CDCl_3$): δ 12.97 (s, 1H, N*H*), 7.42 (d, $^3J_{HH}$ = 9 Hz, 4H, aryl C*H*), 7.11 (d, $^3J_{HH}$ = 8 Hz, 8H, aryl C*H*), 7.04 – 6.92 (m, 12H, aryl C*H*), 2.34 (s, 12H, CH_3).^{13}C{^{1}H} NMR (101 MHz, $CDCl_3$): δ 149.0, 144.4, 140.6, 134.0, 130.3, 126.2, 125.5, 121.7, 120.7, 115.4, 21.0. UV-vis (toluene): λ_{max} = 546 nm (ε = 23200 M^{-1} cm^{-1}). Mass spec. (ESI, +ve mode): exact mass calculated for [C42H38N7]+, [M+H]+ = 640.3189; exact mass found: 640.3183; difference = −0.9 ppm.

Synthesis of BDF dye **3**

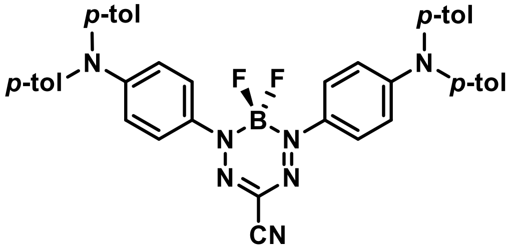

To a solution of formazan 2 (2.00 g, 3.13 mmol) in toluene (100 mL), triethylamine (1.31 mL, 9.38 mmol) was added dropwise. The solution was stirred for 10 min before boron trifluoride diethyl etherate (1.93 mL, 15.6 mmol) was added. Then, the reaction mixture was heated at 80 °C for 16 h. After cooling to 20 °C, deionized water (20 mL) was added to quench any unreacted boron-containing compounds. The organics were then extracted with ethyl acetate (100 mL) and washed with deionized water (3 × 150 mL). After drying over $MgSO_4$, the volatiles were removed *in vacuo*. The resulting residue was purified by flash chromatography (silica gel, hexane: ethyl acetate = 4:1, R_f = 0.54) to yield the product as a dark purple solid. Yield = 1.11 g, 52%. ^{1}H NMR (600 MHz, $CDCl_3$): δ 7.76–7.74 (m, 4H, aryl C*H*), 7.14 (d, $^3J_{HH}$ = 6 Hz, 8H, aryl C*H*), 7.08–7.06 (m, 8H, aryl C*H*), 6.97–6.95 (m, 4H, aryl C*H*), 2.37 (s, 12H, CH_3). ^{13}C{^{1}H} NMR (151 MHz, $CDCl_3$): δ 150.5, 143.6, 136.4, 135.1, 130.4, 126.8, 126.2, 124.2, 119.8, 115.2, 21.1. ^{11}B NMR (193 MHz, $CDCl_3$): δ –0.5 (t, $^1J_{BF}$ = 31 Hz). ^{19}F NMR (565 MHz, $CDCl_3$): δ –136.4 (q, $^1J_{FB}$ = 28 Hz). UV-vis (toluene): λ_{max} = 785 nm (ε = 47800 M^{-1} cm^{-1}). Mass spec. (ESI, +ve mode): exact mass calculated for $[C_{42}H_{37}N_7BF_2]^+$, $[M+H]^+$ = 688.3172; exact mass found: 688.3166; difference = –0.6 ppm.

Computational Methodology All electronic structure calculations were carried out with the *Gaussian* program[2] using the 6-311+g(d) basis set and M06 density functional.[3,4] All structures were confirmed to be at an energetic minima through vibrational analysis. Ground-state and excited-state TDDFT calculations of all

Materials for Formulation and characterization of BDF-NE Glyceryl trioctanoate were obtained from Sigma Aldich (USA). 18:0 PEG2000 PE (1,2-distearoyl-sn-glycero-3-phosphoethanolamine-N-[methoxy(polyethylene glycol)-2000]) and DSPC (1,2-distearoyl-sn-glycero-3-phosphocholine) were purchased from Avanti Polar Lipids (USA). All other solvents and reagents were of reagent grade and obtained from Sigma Aldrich (USA) and were of reagent grade. Water was purified with Milli-Q Plus 185

water purification system (Millipore, Bedford, MA). Absorbance and emission spectra were collected on a UV–vis spectrophotometer Cary 50 (Agilent). The fluorescence spectrum was recorded using a SENSE spectrometer (Sarspec, Portugal). An 808 nm laser (MDL-XF-808-5W, CNI Laser) was employed as the excitation source. The laser power was set to ~ 2.5 W. Dynamic light scattering (DLS) was used to measure the hydrodynamic diameter and Polydispersity Index (PdI) of the nanoemulsions. The Measurements were performed with a Zetasizer Pro (Malvern Instrument) at room temperature. TEM imaging was performed using Hitachi HT7800 microscope (Hitachi High Technologies) at Nanoscale Biomedical Imaging Facility, The Hospital for Sick Children, Toronto, Canada.

Nanoemulsion formulation Boron difluoride formazanate **3** nanoemulsion (**BDF-NE**) was prepared via a sonication-assisted method. 18:0 PEG2000 PE (31.6 mg) and DSPC (87mg), together with BDF **3** (17.2 mg), were dissolved in 2.0 mL of chloroform and thoroughly mixed. The solvent was then evaporated under a gentle nitrogen stream to form a thin lipid film. Subsequently, Glyceryl trioctanoate (148.2 mg) was added to the lipid film, followed by the addition of 2.0 mL of deionized distilled water (ddH_2O). The resulting mixture was sonicated at 40 °C to promote hydration, after which the temperature was increased to 50 °C and sonication was continued for an additional 60 min. The obtained dispersion was centrifuged at 4000 rpm for 5 min, and the supernatant was filtered through a polyethylene membrane to eliminate precipitates. The resulting nanoemulsion was stored at 4 °C until further characterization.

Oil-free nanoparticle formulation 18:0 PEG2000 PE (2.6 mg), DSPC (13.8 mg), and BDF 3 (1.0 mg) were dissolved in chloroform. The solvent was removed under a gentle stream of nitrogen gas, and the mixture was vacuum-dried for 3 h to remove residual solvent. The resulting lipid film was rehydrated with 1.0 mL of PBS (pH 7.4) and subjected to 10 freeze–thaw cycles using liquid nitrogen and a 65 °C water bath. The suspension was then extruded twice through a polycarbonate membrane (pore size = 100 nm) using a mini-extruder (Avanti Polar Lipids). Particle size and polydispersity were determined by dynamic light scattering (ZS90 Nanosizer, Malvern Instruments). The resulting nanoparticles (**BDF-LP**) were stored at 4 °C until further use.

Photothermal efficacy evaluation in aqueous solution For photothermal measurements in aqueous dispersions, **BDF-NE** was diluted in water to 1.0 mL at a concentration adjusted to give an optical density (OD) of 1.0 at 825 nm. Aliquots were transferred to circular polystyrene wells (1 cm internal diameter). Samples were irradiated with an 825 nm continuous-wave laser (LASERGLOW TECHNOLOGIES) at incident irradiances of 0.2–1.0 W/cm^2. Laser powers were measured with a power meter (THORLABS, SN: S405C, P_{max}= 5W). Temperature was monitored using either an infrared thermal camera (C3-X, FLIR, USA) or a thermocouple module (hypodermic needle microprobe MT-23/3HT, PHYSITEMP INSTRUMENTS; USB-TC01, NATIONAL INSTRUMENTS) logging at 1 Hz. For on/off cycling, **BDF-NE** (20 μM) was subjected to seven irradiation cycles consisting of 9 min laser on followed by 14 min laser off.

Calculation of photothermal conversion efficiency The nanoemulsion solution was dispersed in water and adjusted to give an OD of 1.0 at 825 nm in the measurement geometry. For photothermal measurements, 1 mL of dispersion (or ddH_2O for blanks) was transferred to a circular polystyrene container (1 cm internal diameter). Samples were irradiated with a continuous-wave 825 nm laser delivering a uniform irradiance of 1.0 W/cm^2 at the sample plane (beam area A_{beam} = 0.71 cm^2; thus power I=0.71 W).

Temperature vs time was recorded with a thermocouple module at 1 Hz. For each sample, we recorded a heating segment (laser ON until the temperature approached a plateau) followed by a cooling segment (laser OFF).

Photothermal conversion efficiency (η) calculations were performed based on Roper's report.[5] In this method, they simplified energy balance of system to:

$$\sum_i m_i\, C_{p,i} \frac{dT}{dt} = Q_I + Q_0 - Q_{ext} \qquad \cdots\cdots (1)$$

$\sum mC_p$: Mass and heat capacity of all system components

T: system temperature

Q_I: Heat generated by converting light absorbed by nanoparticles into thermal energy

Q_0: Heat generated by converting light absorbed by container and solvent into thermal energy

Q_{ext}: heat loss to surroundings

The Q_I is determined with the following equation:

$$Q_I = I(1 - 10^{-A_\lambda})\eta \qquad \cdots\cdots (2)$$

I: incident laser power

A_λ: the absorbance of the nanoparticle at wavelength of λ

η: the efficiency of transducing absorbance to thermal energy

External heat loss modeled as a linear driving force:

$$\mathrm{Q_{ext}} = \mathrm{hS(T - T_{amb})} \qquad \cdots\cdots (3)$$

h: heat transfer coefficient

S: surface area of the container

T_{amb}: ambient temperature of the surroundings

Substitution eq. 3 into the energy balance eq. 1 yields the first order ordinary differential equation given as:

$$\sum_i m_i\, C_{p,i} \frac{dT}{dt} = Q_I + Q_0 - \mathrm{hS(T - T_{amb})} \qquad \cdots\cdots (4)$$

To interpret eq. 4, a dimensionless driving force temperature θ is introduced using the maximum system temperature T_{max}:

$$\theta = \frac{T - T_{amb}}{T_{max} - T_{amb}} \qquad \cdots\cdots (5)$$

And a sample system time constant τ_s:

$$\tau_s = \frac{\sum_i m_i C_{p,i}}{\mathrm{hS}} \quad \cdots\cdots (6)$$

Which is substituted into eq. 4 and rearranged to yield:

$$\frac{d\theta}{dt} = \frac{1}{\tau_s}\left[\frac{Q_I + Q_0}{hS(T_{max} - T_{amb})} - \theta\right] \quad \cdots\cdots (7)$$

To obtain the τ_s, we use the cooling section where laser irradiation is ceased.
With the laser off, internal sources vanish (Q_I = 0, Q_0 = 0), giving:

$$dt = -\tau_s \frac{d\theta}{\theta} \quad \cdots\cdots (7)$$

And integrating eq. 7 yield:

$$t = -\tau_s \ln \theta \quad \cdots\cdots (8)$$

Thus, a linear fit of lnθ vs. t over the linear region yields slope = $-1/\tau_s \rightarrow \tau_s$

In out experiment, τ_s was determined to be 340.3s by applying the linear time data from the cooling period vs negative natural logarithm of driving force temperature.
In addition, the m is 1g and C is 4.18 J/g°C. Thus, according to eq. 6, hS is deduced to be 12.28 mW/°C.
With hA known, Q_0 was determined as 193.2 mW from the blank (solvent-only) heating experiment by evaluating the steady-state temperature rise during continuous irradiation. At the steady-state, without any nanoparticle involved:

$$\mathrm{Q_0 = hS(T - T_{amb})} \quad \cdots\cdots (9)$$

With knowing hS, τ_s and Q_0, we can calculate η from eq. 2. At the plateau, Q_I + Q_0 = Q_{ext}.

$$\eta = \frac{Q_I}{I(1-10^{-A_\lambda})} = \frac{Q_{ext} - Q_0}{I(1-10^{-A_\lambda})} = \frac{\mathrm{hS}(\mathrm{T}_{max} - \mathrm{T_{amb}}) - Q_0}{I(1-10^{-A_\lambda})} \quad \cdots\cdots (10)$$

η of BF1 NE calculated as 0.668 (66.8%).

Absolute photoluminescence quantum yield (PLQY) measurements

Absolute PLQY measurements were conducted using a Thorlabs PM400 optical power meter equipped with a Thorlabs S122C silicon photodiode sensor (spectral response range: 700–1800 nm) and an integrating sphere (model JY-TFIOS-84, Jingyi Photoelectric Technology Co.). The excitation source was an 808 nm CW laser (MDL-XF-808-5W, CNI Laser) coupled directly into the integrating sphere. At the

detector port of the integrating sphere, an 800 ± 10 nm bandpass filter was used for excitation power measurements, and a 1000 nm long-pass filter was used for NIR-II emission measurements.

Measurements were performed in two configurations: (1) a blank measurement using a quartz cuvette containing water only, in which the excitation power entering the sphere (P_{ex},blank) and any background emission (P_{em},blank) were recorded; and (2) a sample measurement using BDF-NE or BDF-Lipid dispersed in water (20 μM) in the same cuvette geometry, in which the transmitted excitation power (P_{ex},sample) and the NIR-II emission power (P_{em},sample) were recorded. The power meter wavelength was set to 808 nm for excitation measurements and to 1060 nm for emission measurements; the latter was used as a representative wavelength for responsivity correction within the NIR-II emission band. The number of absorbed and emitted photons was calculated from the measured optical powers using the relation N = Pλ/hc, where P is the measured power, λ is the set wavelength, h is Planck's constant, and c is the speed of light. The PLQY was then calculated as:

$$\mathrm{PLQY} = \frac{\lambda_{em}(P_{em,sample} - P_{em,blank})}{\lambda_{ex}(P_{ex,sample} - P_{ex,blank})}$$

where λ_{em} = 1060 nm and λ_{ex} = 808 nm.

PA phantom imaging PA signal intensity was evaluated using a tubing phantom model. Polyethylene tubing (diameter 0.38mm) were filled with water or aqueous solutions of **BDF-NE** at varying concentrations (20, 40, 60, 80 and 100 μM). Photoacoustic imaging was performed using Vevo LAZR-X imaging system (VisualSonics), and imaging conditions were as follows. Ultrasound gain = 18 dB; Photoacoustic gain = 35 dB; time gain compensation = uniform; spectral range = 680–970 nm; data interval = 1 nm.

***In vivo* PA imaging and fluorescence imaging** All animal studies were approved and conducted in compliance with the University Health Network Animal Resources Centre guidelines. Experiments adhered with all relevant institutional, provincial, and federal requirements. For the generation of subcutaneous KB tumor model, athymic female nude mice under general anesthesia (2 v/v% isoflurane in oxygen) were inoculated with 1.5×10^{6} KB cells in a 1:1 mixture of FBS free media and Matrigel (total volume of 100 μL) into the right leg. Tumor growth was monitored using electronic calipers.

When the KB tumors reached a size of 300 mm^3, pre-injection PA imaging was performed prior to nanoparticle injection after mice were safely anesthetized with vaporized isoflurane. Mice were then injected intravenously with **BDF-NE** (6.8 mg/kg based on **3**, 200 μL per mouse) and conducted for tumor PA imaging at different time points (1, 3, 6, 9, 14, 24, and 48 hours). PA imaging were performed using a Vevo LAZR2100, and imaging conditions were as follows: ultrasound gain = 15 dB; photoacoustic gain = 37 dB; time gain compensation = uniform, spectral range = 680 – 970 nm; data interval = 1 nm.

***In vivo* NIR-II fluorescence imaging study** *In vivo* fluorescence biodistribution was evaluated in six athymic nude mice bearing subcutaneous tumors (KB cell line). One mouse served as a naïve control, and five mice received an intravenous (tail vein) injection of **BDF-NE** at a dose of 6.8 mg/kg (1.24 mM, 200 µL per mouse). All animal procedures were conducted in accordance with the Guidelines for the Care and Use of Laboratory Animals (National Research Council of Canada).

Fluorescence imaging was performed using an IR VIVO SynIRgy™ system (Photon etc., Montreal, Canada) equipped with an InGaAs Alizé™ 1.7 camera. Excitation was provided by a 785 nm laser at 23 mW/cm², and fluorescence emission was sequentially collected using 850 nm, 1000 nm, and 1250 nm long-pass (LP) filters with exposure times of 1 ms, 2 ms, and 20 ms, respectively.

For all **BDF-NE** treated mice, pre-injection whole-body fluorescence images were acquired to establish baseline signal levels. Following intravenous administration, whole-body fluorescence images were collected at 1, 3, 6, and 24 hours post-injection to assess temporal biodistribution and tumor accumulation. At 24 hours post-injection, both **BDF-NE** treated and naïve control mice were euthanized. Major organs, including tumor, heart, liver, spleen, kidneys, lungs, skin, muscle, brain, and small intestine, were excised and imaged ex vivo using the same acquisition parameters. Fluorescence intensity was quantified from manually defined regions of interest (ROIs) for each organ. Background fluorescence was subtracted from each ROI to correct for non-specific signal. Both total and mean fluorescence intensities were calculated to enable quantitative comparison of **BDF-NE** accumulation across tissues, with emphasis on tumor uptake relative to naïve controls.

***In vivo* PTT study** All animal studies were approved and conducted in compliance with the University Health Network Animal Resources Centre guidelines. The KB tumor model was generated using the same method as described above. Once the tumors reached 100 mm^3, the animals were randomly divided into different groups and **BDF-NE** 6.8 mg/kg (200 µL per mouse) was administered by tail vein injection to the injection only group and PTT group. The mice in PTT and Laser only groups received laser irradiation (825 nm, 1 W/cm^2) on their tumor site for 4 minutes at 6 hours post injection. During laser irradiation, the temperature of the tumor site was monitored at 10-second intervals until 1 min and 30-second intervals until 5 min using a thermal imaging camera and implanted thermocouple. Tumor volume and body weight of each animal were measured every 2–3 days. A humane endpoint was set when tumor volume exceeded 1000 mm^3 or severe ulceration was observed. Tumor volume was calculated from caliper measurements using Eq. 11.

$$Tumor\ volume = \frac{(Width^2 \times Length)}{2} \qquad \cdots\cdots (11)$$

Statistical analysis

The data of size distribution, mean PA average at 780nm, temperature increase of the tumor sites and body weight, average fluorescence intensity from tumor and average fluorescence intensity change are shown as mean ± standard deviation (SD). For tumor fluorescence intensity at different time points, one-way ANOVA with Dunnett's test was performed using GraphPad Prism. To determine the statistical significance in survival study, Logrank test for trend was used. Statistical analysis was performed using GraphPad Prism.

Supplementary Figures and Tables

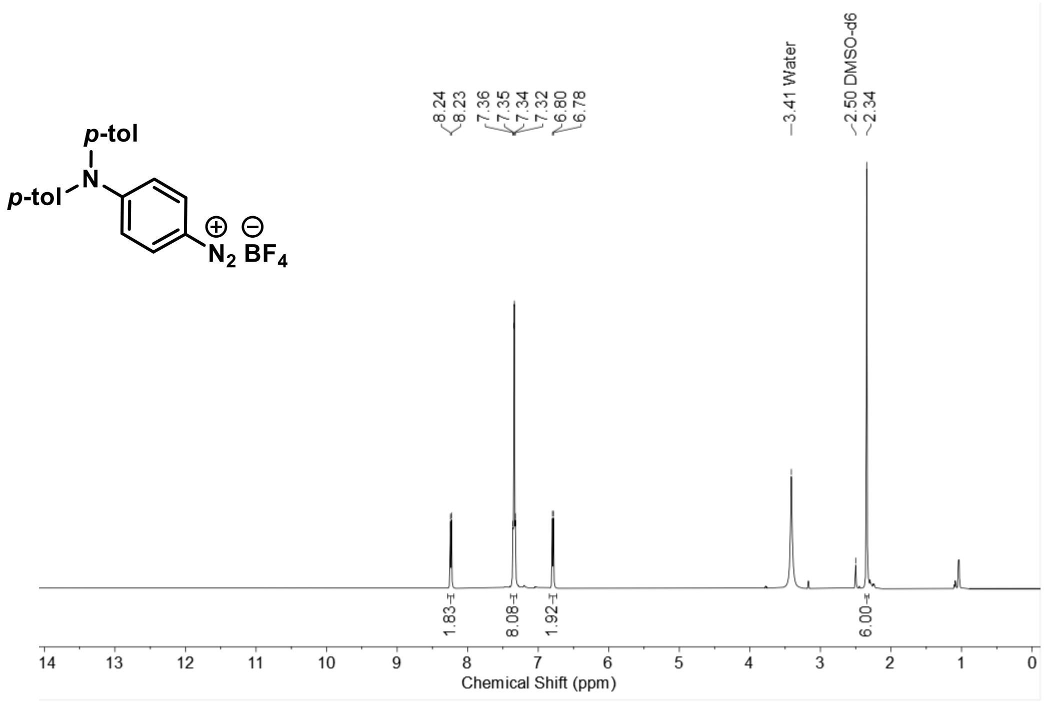

Figure S1. ^{1}H NMR spectrum of diazonium tetrafluoroborate **1** recorded in DMSO-*d*$_6$.

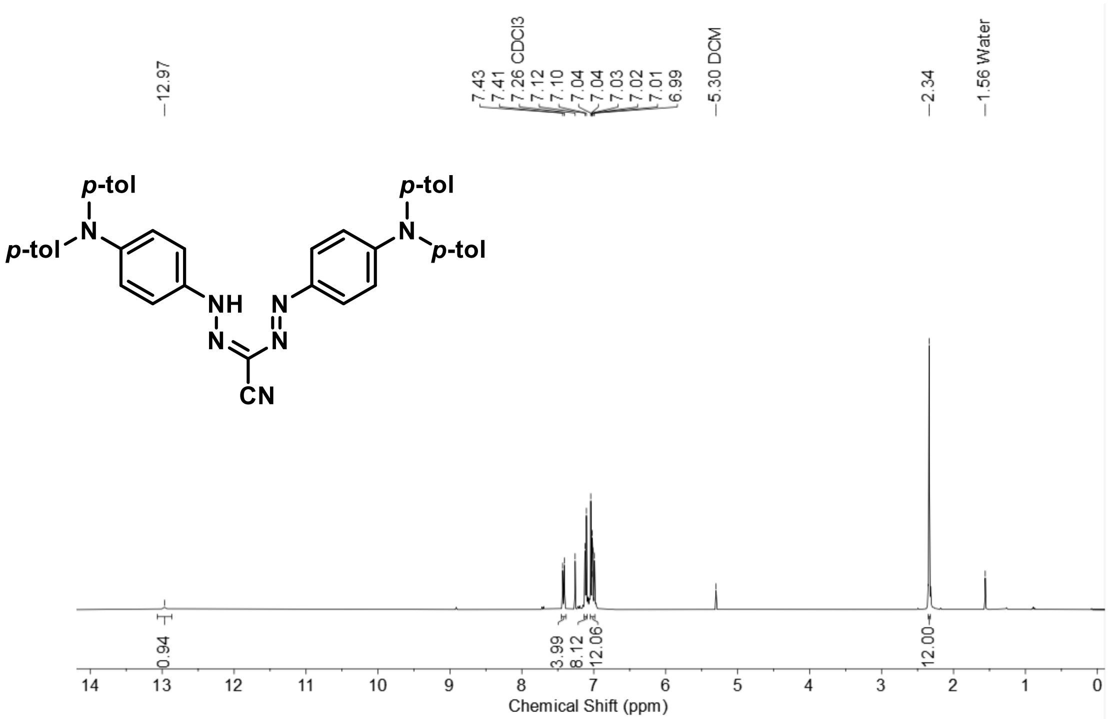

Figure S2. ^{1}H NMR spectrum of formazan **2** recorded in $CDCl_3$.

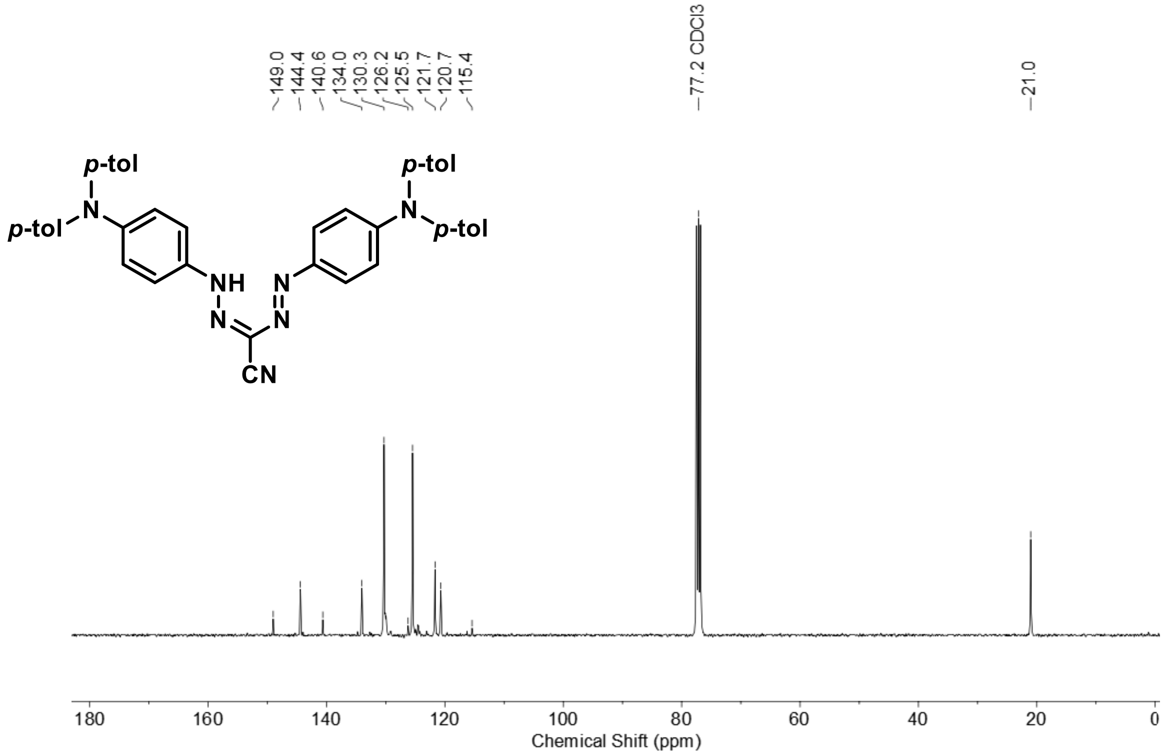

Figure S3. $^{13}C\{^{1}H\}$ NMR spectrum of formazan **2** recorded in $CDCl_3$.

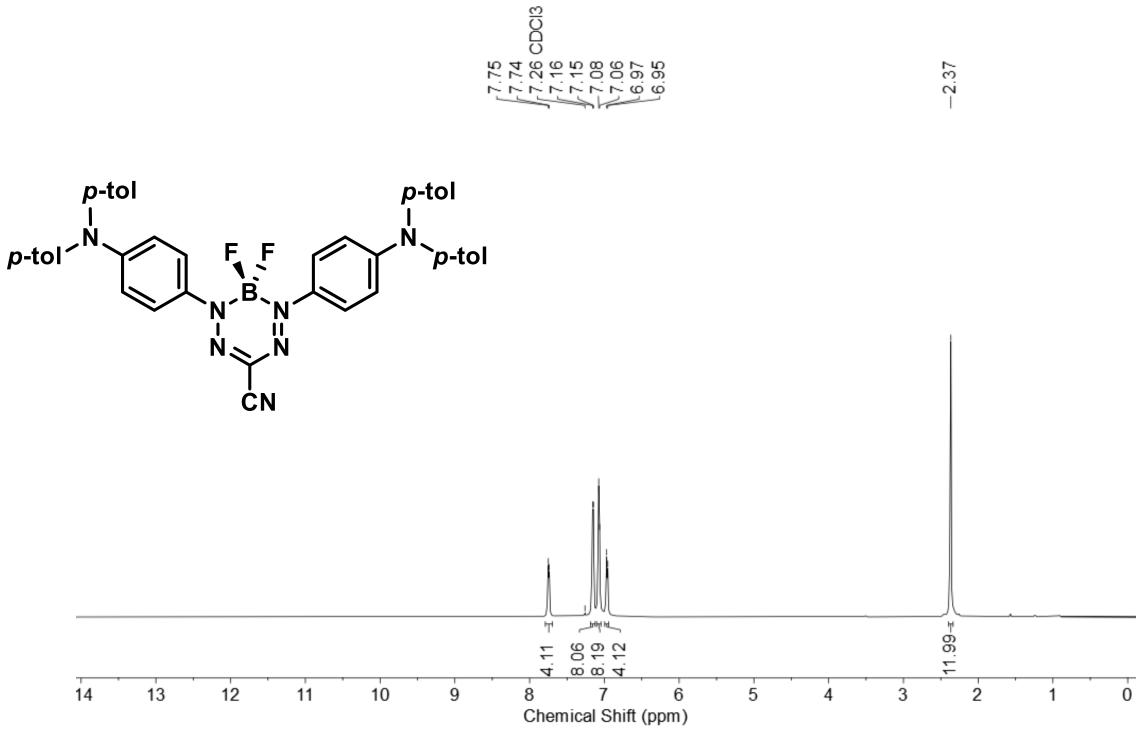

Figure S4. ^{1}H NMR spectrum of boron difluoride formazanate **3** recorded in $CDCl_3$.

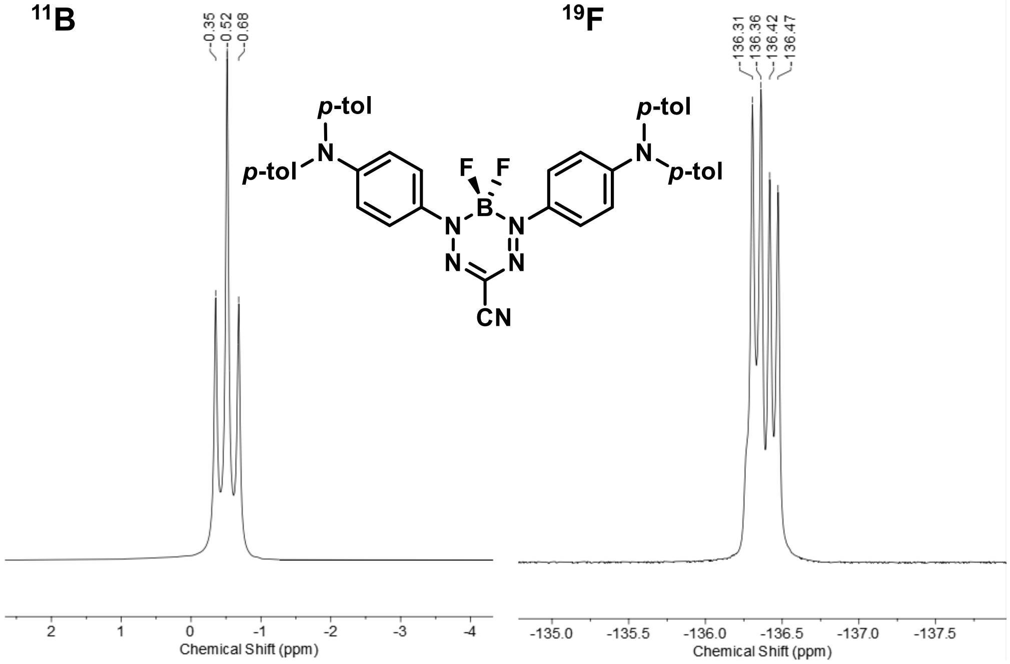

Figure S5. ^{11}B and ^{19}F NMR spectra of boron difluoride formazanate **3** recorded in $CDCl_3$.

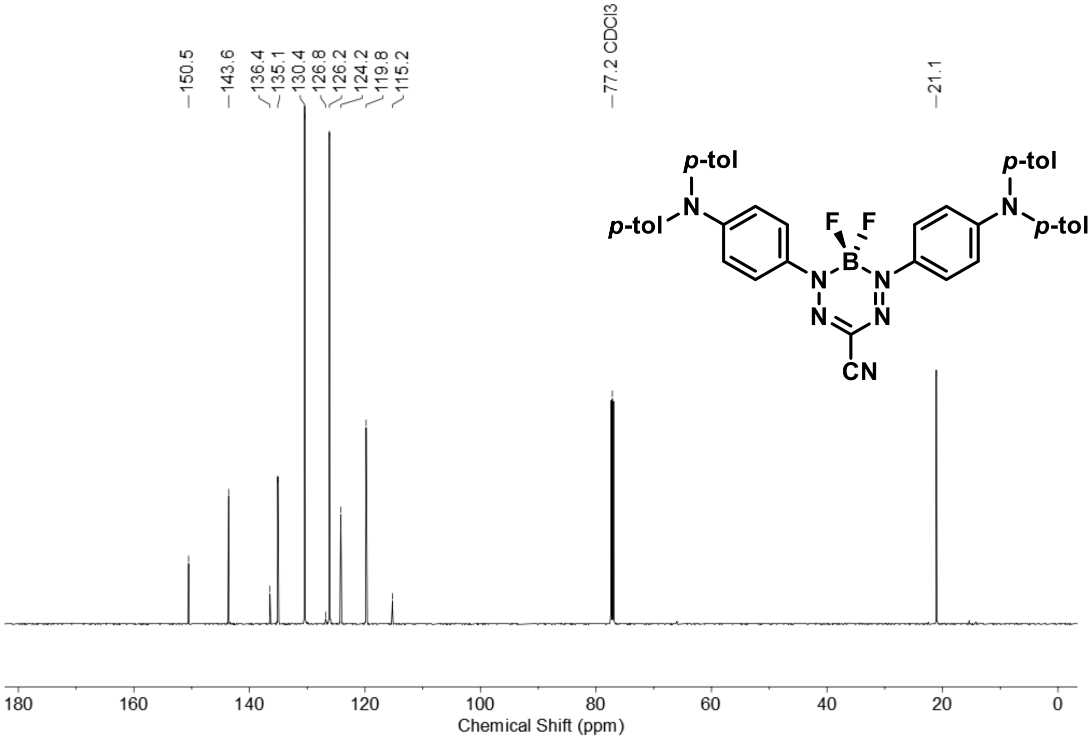

Figure S6. $^{13}C\{^{1}H\}$ NMR spectrum of boron difluoride formazanate **3** recorded in $CDCl_3$.

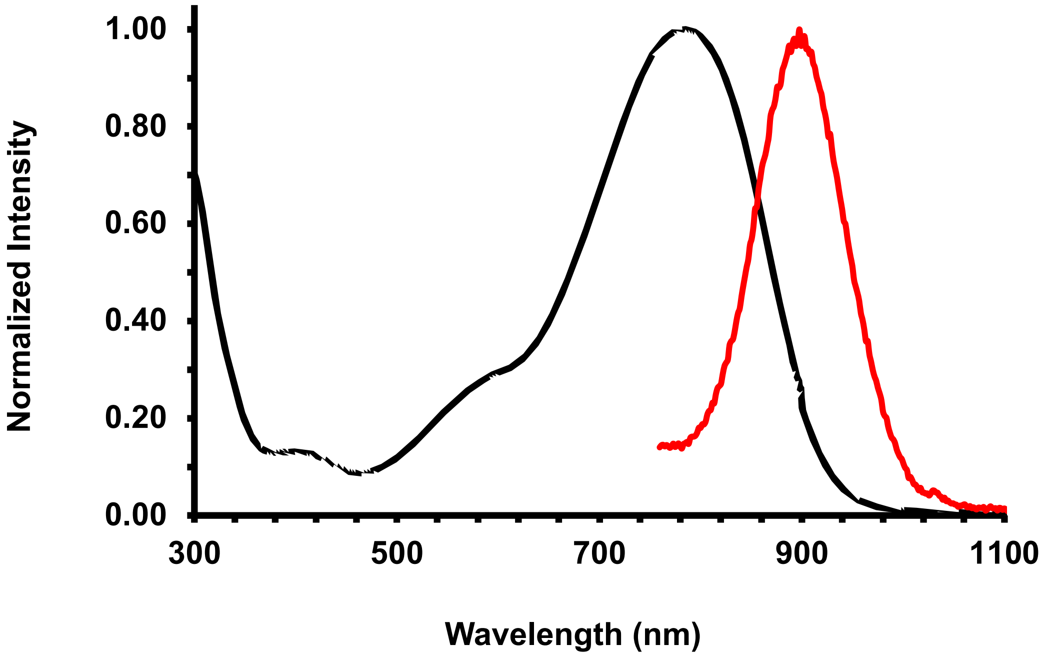

Figure S7. UV-vis absorption spectrum (black) and photoluminescent spectrum (red) of 5 μM boron difluoride formazanate **3** in toluene.

LUMO

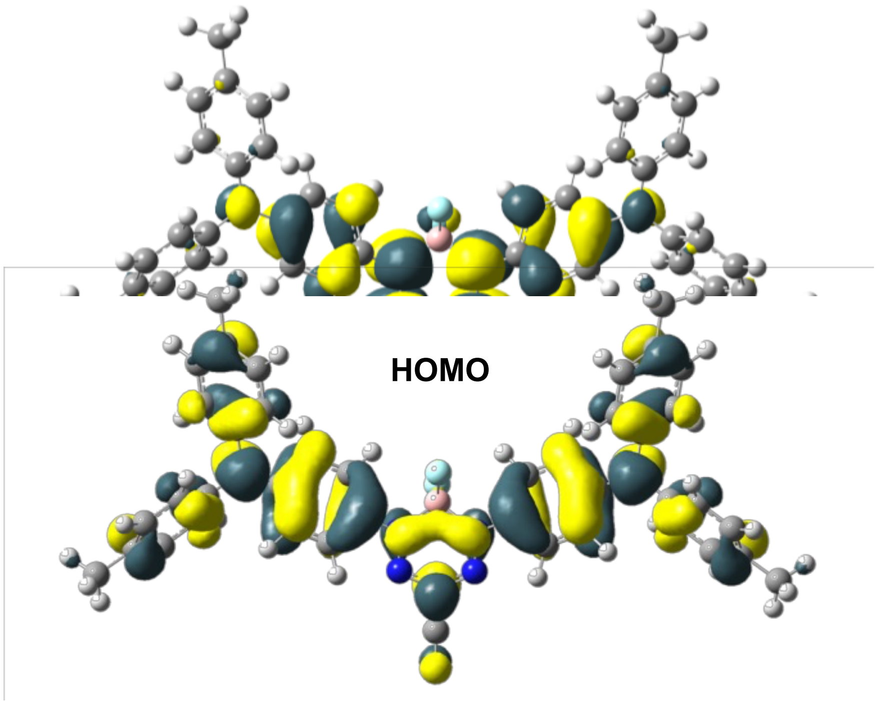

Figure S8. Frontier molecular orbitals computed for boron difluoride formazanate **3** using DFT M06/6-311+g(d) scrf=(PCM, solvent=toluene) method. compounds were calculated using polarizable continuum model (PCM) of implicit solvation.

Table S1. Maximum absorption/emission wavelengths of boron difluoride formazante **3** calculated using the TDDFT M06/6-311+g(d) scrf=(PCM, solvent=toluene) method compared to experimental values in toluene.

	Experimental		Theoretical	
	**λ_{abs} (nm)**	**λ_{PL} (nm)**	**λ_{abs} (nm)**	**λ_{PL} (nm)**
BF_2 Formazanate **3**	785	898	747	957

Optimized Atomic Coordinates

td=(root=1) M06/6-311+g(d) scrf=(PCM, solvent=toluene) int=grid=superfine

Compound **3**, ground-state structure (C_1 symmetry)
SCF Done: E(RM06) = -2228.97626948

0 1

C	4.06410	0.52143	-0.82852
C	5.03064	-0.11500	-0.03619
C	4.69607	-1.34132	0.56168
C	3.45703	-1.90623	0.36655
C	2.50288	-1.26682	-0.42946
C	2.82076	-0.04075	-1.01928
H	4.30502	1.46156	-1.31696
H	5.41841	-1.83981	1.20252
H	3.20472	-2.84639	0.84803
H	2.10632	0.46042	-1.66395
N	6.28621	0.45166	0.15745
C	7.42126	-0.36457	0.41299
C	7.69755	-1.46760	-0.39069
C	8.28365	-0.05683	1.46159
C	8.80895	-2.25444	-0.13482
H	7.03143	-1.70929	-1.21658
C	9.40011	-0.84280	1.69785
H	8.07174	0.80461	2.09209
C	9.68398	-1.95558	0.90805
H	9.00898	-3.11675	-0.76981
H	10.06328	-0.59244	2.52522
C	6.47376	1.85820	0.08684
C	5.63946	2.72261	0.79179
C	7.51718	2.38869	-0.66643
C	5.83878	4.09179	0.72391
H	4.83264	2.31383	1.39690
C	7.71503	3.75957	-0.71418
H	8.17858	1.71742	-1.21077
C	6.87734	4.63735	-0.02922
H	5.17953	4.75469	1.28335
H	8.53744	4.15890	-1.30665
C	0.00005	-3.73065	-0.55990
C	-2.50283	-1.26688	-0.42944
C	-2.82077	-0.04086	-1.01935
C	-3.45694	-1.90625	0.36668
C	-4.06414	0.52127	-0.82861

H	-2.10637	0.46027	-1.66409
C	-4.69599	-1.34135	0.56181
H	-3.20457	-2.84635	0.84824
C	-5.03062	-0.11512	-0.03619
H	-4.30510	1.46136	-1.31712
H	-5.41827	-1.83979	1.20273
N	-6.28620	0.45154	0.15744
C	-7.42124	-0.36464	0.41313
C	-7.69751	-1.46788	-0.39021
C	-8.28369	-0.05659	1.46165
C	-8.80895	-2.25467	-0.13414
H	-7.03137	-1.70982	-1.21601
C	-9.40014	-0.84246	1.69808
H	-8.07179	0.80504	2.09190
C	-9.68399	-1.95549	0.90858
H	-9.00892	-3.11716	-0.76889
H	-10.06335	-0.59187	2.52535
C	-6.47375	1.85807	0.08661
C	-5.63941	2.72260	0.79129
C	-7.51725	2.38842	-0.66672
C	-5.83872	4.09181	0.72314
H	-4.83253	2.31395	1.39641
C	-7.71509	3.75925	-0.71472
H	-8.17868	1.71704	-1.21088
C	-6.87732	4.63720	-0.02999
H	-5.17939	4.75479	1.28237
H	-8.53755	4.15849	-1.30721
N	1.24724	-1.86292	-0.65965
N	1.20012	-3.14745	-0.53306
N	-1.24718	-1.86295	-0.65963
N	-1.20003	-3.14749	-0.53303
B	0.00001	-1.01360	-1.10628
F	0.00000	-0.89123	-2.48287
F	-0.00001	0.20359	-0.47420
C	0.00007	-5.15987	-0.47659
N	0.00010	-6.31151	-0.41714
C	7.07139	6.11895	-0.11624
H	8.11926	6.38066	-0.29531
H	6.48751	6.54859	-0.94016
H	6.74957	6.62381	0.80058
C	10.90422	-2.78634	1.15554
H	10.75788	-3.82491	0.84178
H	11.76750	-2.40099	0.59821
H	11.18295	-2.79018	2.21441
C	-10.90431	-2.78602	1.15649
H	-11.17906	-2.79480	2.21639
H	-11.76924	-2.39677	0.60446
H	-10.76042	-3.82317	0.83702
C	-7.07167	6.11872	-0.11759
H	-6.49531	6.54682	-0.94759
H	-8.12106	6.38048	-0.28759
H	-6.74140	6.62495	0.79543

M06/6-311+g(d) scrf=(solvent=toluene,pcm) TD(NStates=1,Root=1) int=grid=superfine

Compound **3**, excited-state geometry (C_1 symmetry)
GS energy = -2228.98371206
ES energy = -2228.93611570

0 1

C	4.02617975	0.58232295	-0.45223444
C	5.14432129	-0.10023134	0.03467314
C	4.94783775	-1.29364650	0.73558812
C	3.67931274	-1.78986301	0.94198344
C	2.55474747	-1.10277131	0.45853341
C	2.75317741	0.09585982	-0.24376804
H	4.16347231	1.50373000	-1.01344276
H	5.80737782	-1.82982801	1.13173382
H	3.53736944	-2.71044331	1.49658821
H	1.90882859	0.64209657	-0.64670573
N	6.44299543	0.40368288	-0.17768958
C	7.48816039	-0.47695742	-0.51984125
C	7.26253620	-1.53370837	-1.40596404
C	8.76290166	-0.31747421	0.03008263
C	8.28893469	-2.40275847	-1.72780091
H	6.27524767	-1.66488252	-1.84292439
C	9.78063466	-1.19049316	-0.30860133
H	8.94409412	0.48833365	0.73781638
C	9.56839923	-2.24858357	-1.19306643
H	8.09569355	-3.21913678	-2.42247720
H	10.76500272	-1.05892479	0.13901004
C	6.69189410	1.78249592	-0.04094144
C	6.06908190	2.51889466	0.97144381
C	7.56315931	2.43963237	-0.91394169
C	6.31344236	3.87378134	1.09702333
H	5.40099097	2.01545639	1.66640355
C	7.80301689	3.79410213	-0.76915283
H	8.04497816	1.88005868	-1.71240943
C	7.18192361	4.54101049	0.23210253
H	5.82736802	4.42942068	1.89801124
H	8.48119399	4.29065078	-1.46197010
C	0.00002028	-3.27105680	1.51539845
C	-2.55470912	-1.10283285	0.45841429
C	-2.75313533	0.09580930	-0.24387520
C	-3.67928495	-1.78991075	0.94187027
C	-4.02612885	0.58229186	-0.45232051
H	-1.90878568	0.64203444	-0.64682721
C	-4.94780079	-1.29368735	0.73548137
H	-3.53734881	-2.71048842	1.49648321
C	-5.14429127	-0.10025275	0.03457711
H	-4.16340409	1.50370660	-1.01351914
H	-5.80733681	-1.82986462	1.13163881
N	-6.44293499	0.40367779	-0.17774997
C	-7.48822739	-0.47695585	-0.51959569
C	-7.26287096	-1.53378662	-1.40565615
C	-8.76284747	-0.31732566	0.03058777
C	-8.28940640	-2.40282286	-1.72718879
H	-6.27568593	-1.66507381	-1.84281790
C	-9.78069932	-1.19030425	-0.30778683
H	-8.94382842	0.48858072	0.73826510
C	-9.56872563	-2.24851202	-1.19219943

H	-8.09634915	-3.21927778	-2.42182242
H	-10.76496943	-1.05863495	0.14001697
C	-6.69176102	1.78255256	-0.04132737
C	-6.06897125	2.51913612	0.97092646
C	-7.56295790	2.43952918	-0.91450452
C	-6.31327625	3.87406523	1.09620324
H	-5.40093017	2.01581742	1.66602215
C	-7.80277399	3.79404234	-0.77001431
H	-8.04477802	1.87979304	-1.71285918
C	-7.18169374	4.54114067	0.23110518
H	-5.82721313	4.42985601	1.89709319
H	-8.48091189	4.29046663	-1.46295998
N	1.26284147	-1.59914255	0.67042798
N	1.20753668	-2.77536420	1.27029477
N	-1.26282045	-1.59921226	0.67031931
N	-1.20751736	-2.77541938	1.27017709
B	0.00002421	-0.79863499	0.21138454
F	0.00007499	-0.67660632	-1.17476857
F	-0.00005037	0.45657757	0.80707497
C	0.00000303	-4.55482604	2.16153012
N	0.00017833	-5.58502866	2.68012024
C	7.41651323	6.01196595	0.36036684
H	8.38747263	6.30555873	-0.05096831
H	6.65130626	6.58390046	-0.18031612
H	7.37790832	6.33819457	1.40499765
C	10.68017211	-3.17323877	-1.57300942
H	10.31162910	-4.17923344	-1.79805749
H	11.20399604	-2.81627941	-2.46924454
H	11.42761185	-3.25618182	-0.77739079
C	-10.68075463	-3.17303947	-1.57171776
H	-11.42618672	-3.25847774	-0.77446582
H	-11.20708803	-2.81420038	-2.46572959
H	-10.31206213	-4.17820695	-1.80014221
C	-7.41623965	6.01213492	0.35903949
H	-6.65115619	6.58392416	-0.18197156
H	-8.38729191	6.30562895	-0.05214968
H	-7.37739666	6.33863410	1.40357636

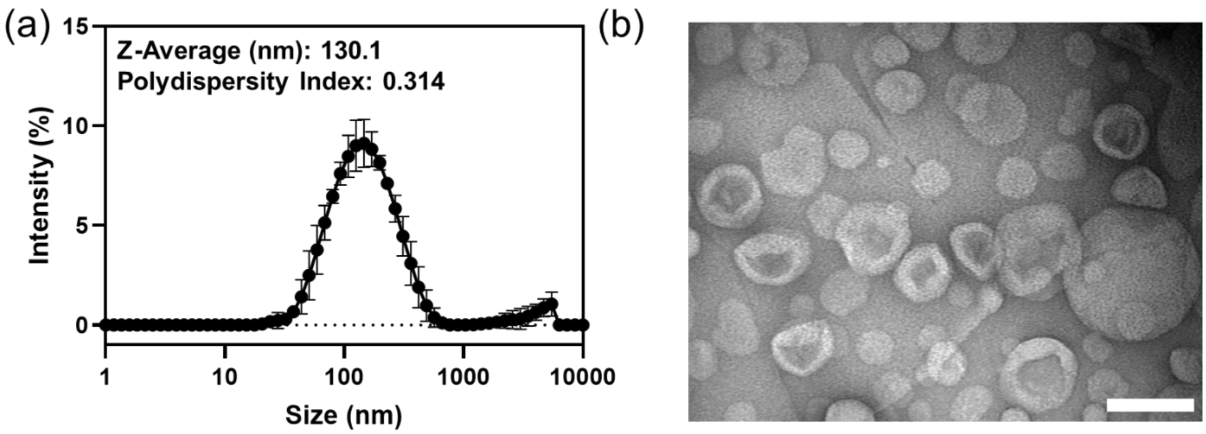

Figure S9. Size distribution of **BDF-NP** measured by dynamic light scattering. (b) Transmission electron microscopy image of **BDF-NP**. Scale bar = 100 nm.

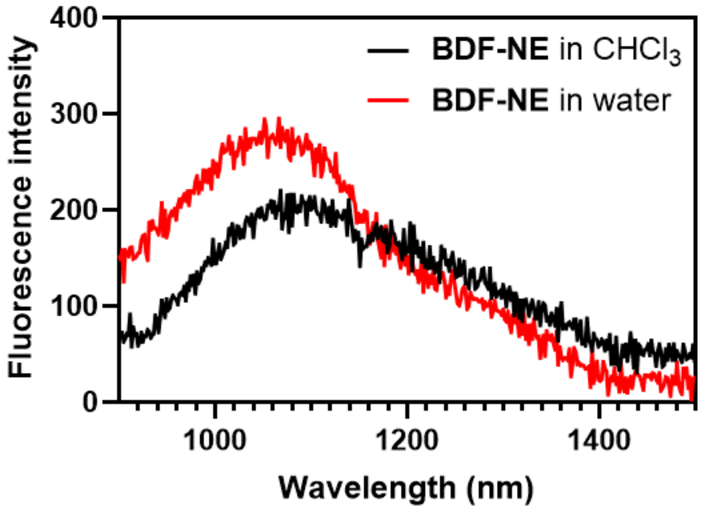

Figure S10. NIR-II fluorescence spectra comparing **BDF-NE** in water (red) and $CHCl_3$ (black). Concentration of **3** was equivalent across all samples. λ_{ex} = 825 nm.

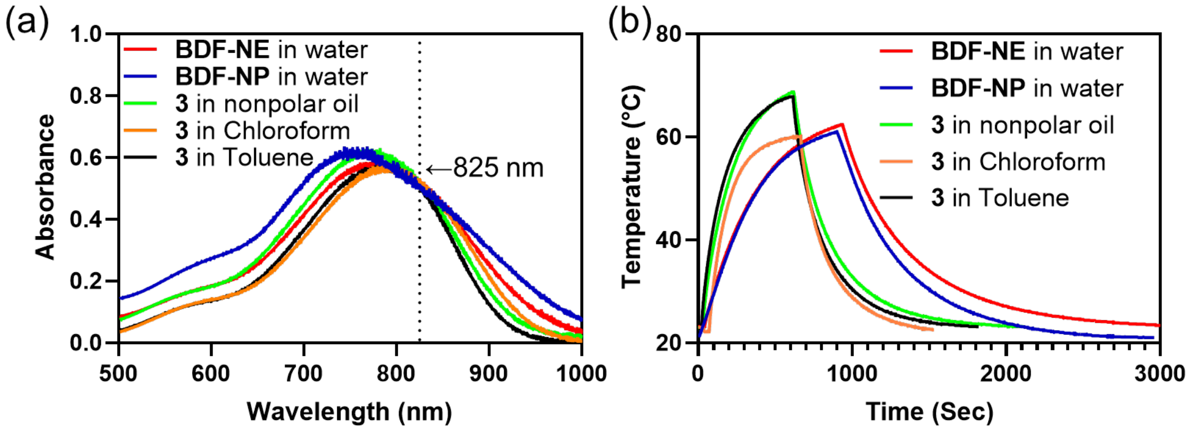

Figure S11. Comparison of optical and photothermal properties of **3** in different environments. (a) UV-vis absorption spectra of **BDF-NE** in water (red), **BDF-NP** in water (blue), **3** in glyceryl trioctanoate (green), **3** in chloroform (orange) and toluene (black) with optical density matched at 825 nm. (b) Temperature profiles of the corresponding solutions under 825 nm laser irradiation (1.0 W cm^{-2}).

Table S2. Comparison of photothermal conversion efficiency (PCE) of reported BDF-based photothermal agents.

Dye name	Structure	NP formulation	λ_{ex}	PCE
Nano-BFF[6]	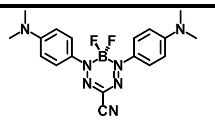	F-127	808 nm<br>1064 nm	28.6%<br>34.3%
BDF1005[7]	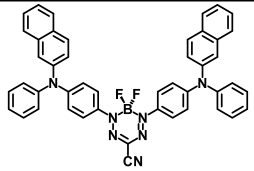	PS-g-PEG	808 nm	39.9%
BDF-Ph[8]	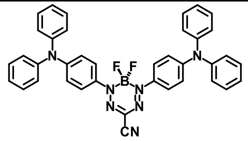	PS-g-PEG	808 nm	40.1%,
TPE-BFF[9]	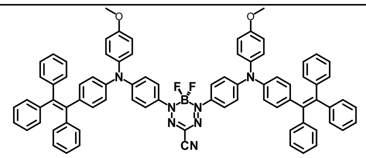	DPPC,<br>DSPE-PEG$_{2000}$-<br>RGD	808 nm	58.5%
NMI-BF$_2$[10]	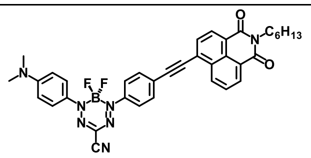	FBS assembly	808 nm	32.5%
BDF-8OMe[11]	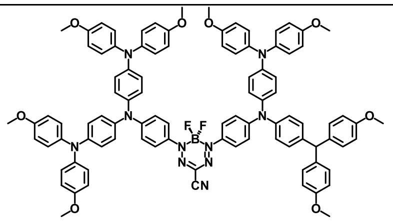	F-127	1064 nm	62.5%
FBDFDPA NP[12]	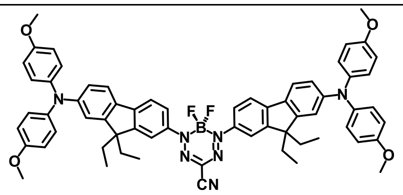	F-127	808 nm<br>1060 nm	49.4%<br>54.4%
BDFTBr[13]	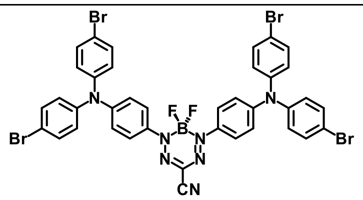	F-127	808 nm	61.3%

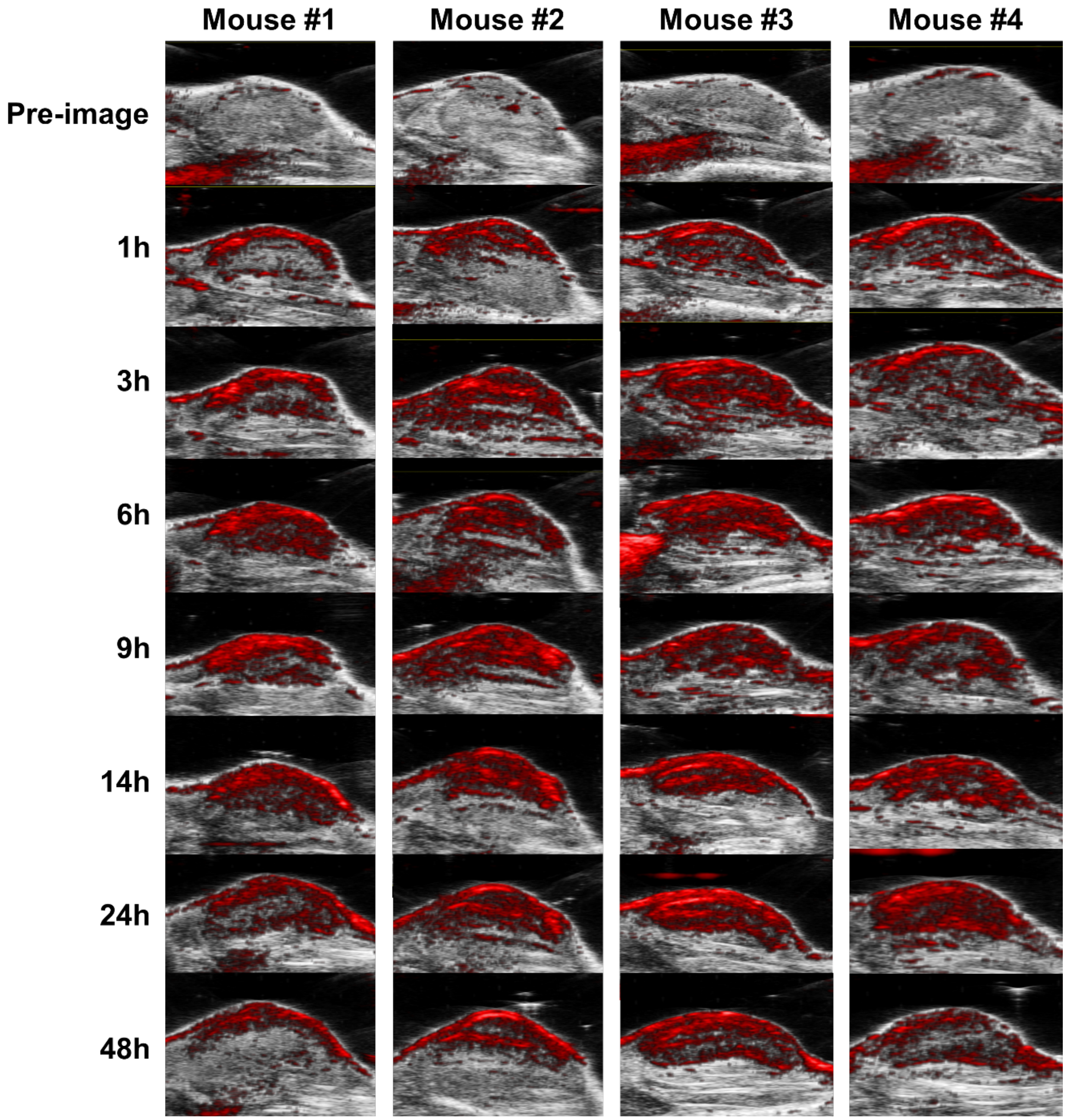

Figure S12. Time-dependent photoacoustic image of tumor sites after intravenous injection of **BDF-NE** (n = 4).

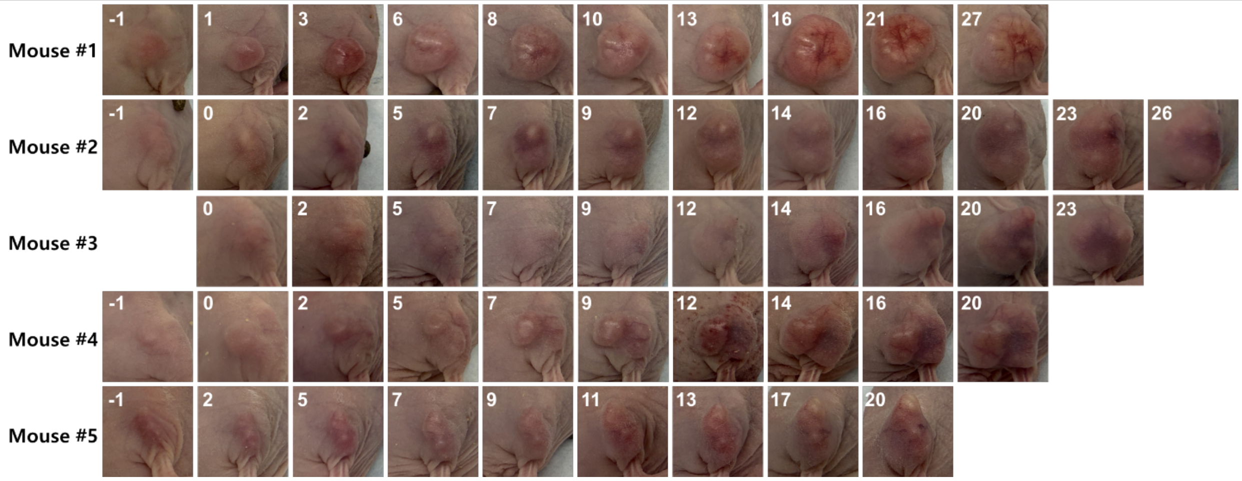

Figure S13. Photographs of tumor sites in mice over time to measure PTT effects (No treatment group).

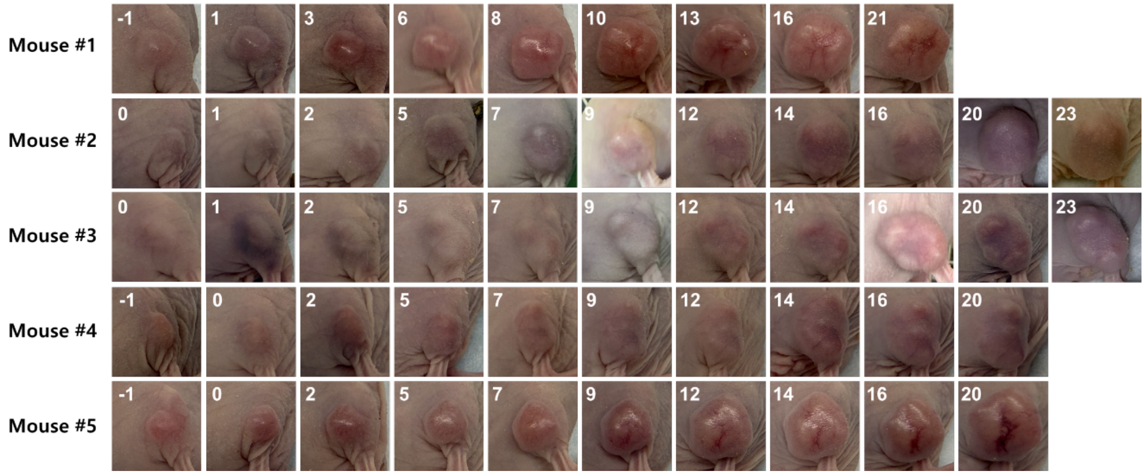

Figure S14. Photographs of tumor sites in mice over time to measure PTT effects (Injection only group).

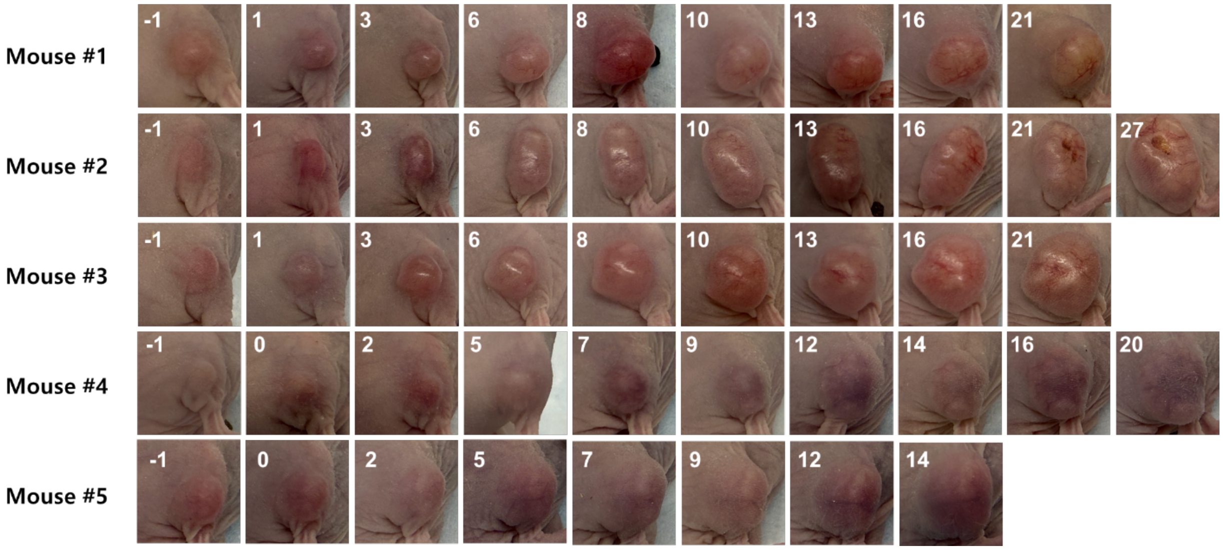

Figure S15. Photographs of tumor sites in mice over time to measure PTT effects (Laser only group).

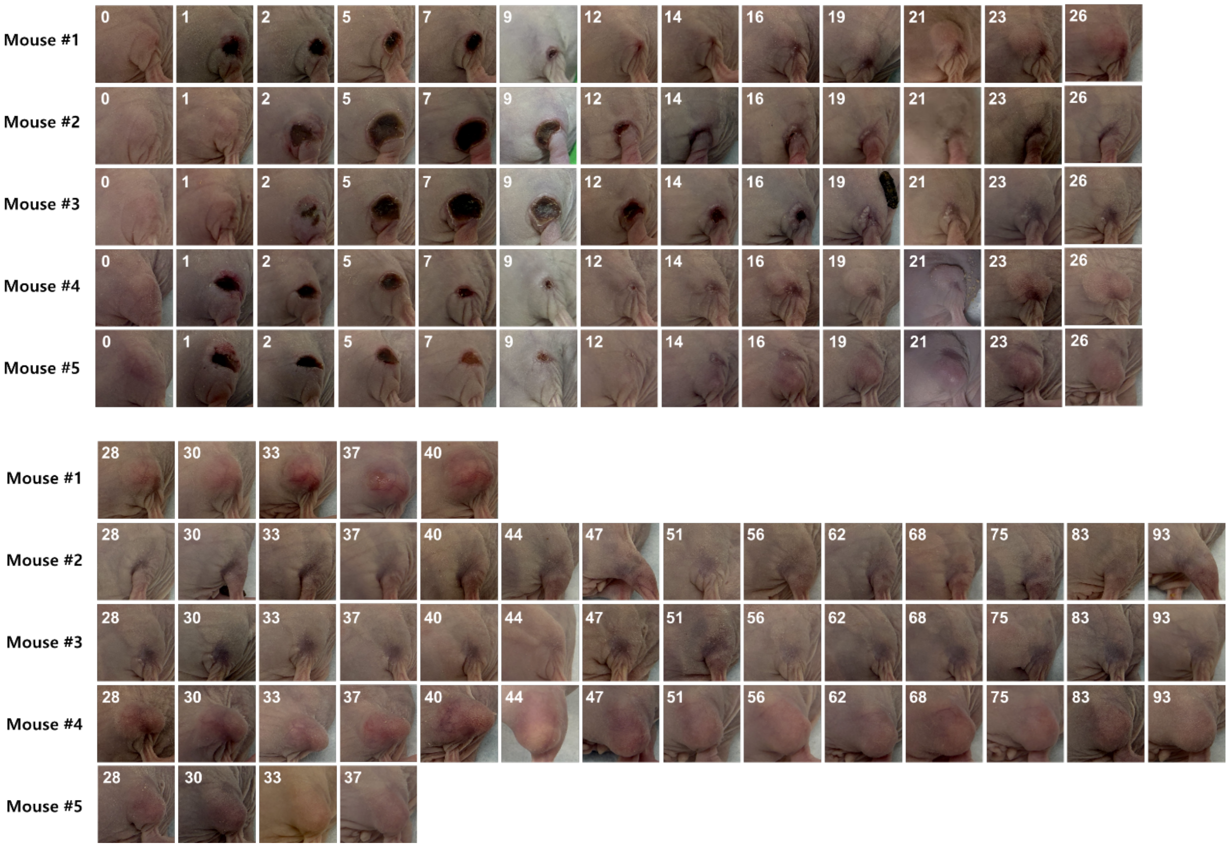

Figure S16. Photographs of tumor sites in mice over time to measure PTT effects (PTT treatment group).

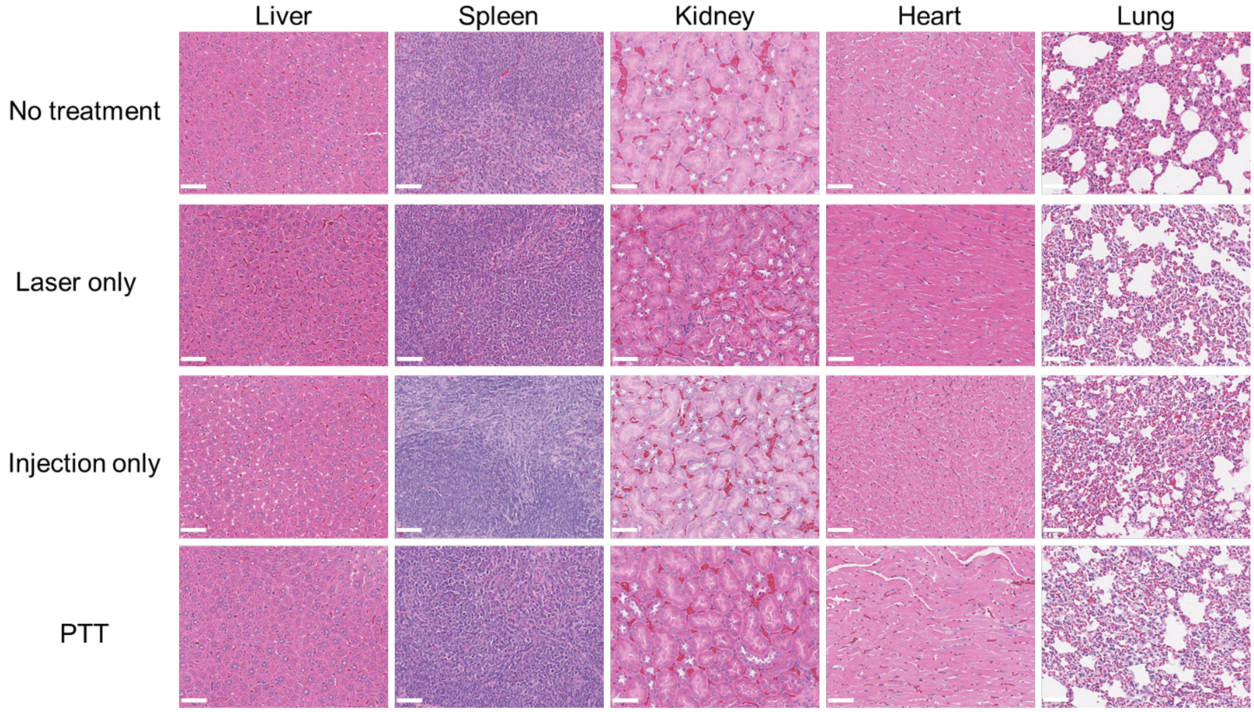

Figure S17. Histopathologic H&E images of organs from mice injected with **BDF-NE** and treated with laser. Mice were sacrificed 48 h after PTT treatment. (Scale bar = 50 µm)

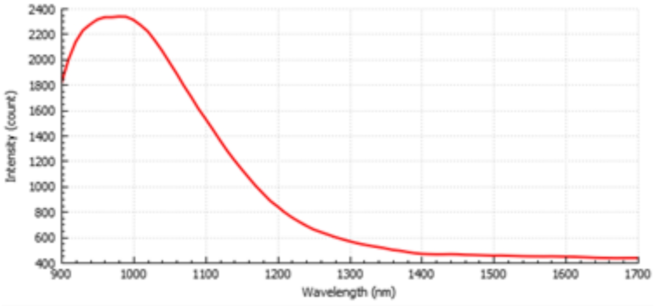

Figure S18. Hyperspectral emission profiling of **BDF-NE** in water (10 µM). Excitation: 785 nm laser at 1.5 W with 50 ND, Camera Exposure time: 0.1s.

References

[1] B. R. Hood, Y. de Coene, C. F. Jones, I. Lopez Poves, N. Deveaux, N. R. Halcovitch, B. Champagne, K. Clays, J. Fielden, “Synthesis and Optical and Nonlinear Optical Properties of Linear and Two-Dimensional Charge Transfer Chromophores Based on Polyoxometalates” *Inorg. Chem.* **2024**, *63*, 24250–24261.

[2] M. J. Frisch, G. W. Trucks, H. B. Schlegel, G. E. Scuseria, M. A. Robb, J. R. Cheeseman, G. Scalmani, V. Barone, G. A. Petersson, H. Nakatsuji, X. Li, M. Caricato, A. V. Marenich, J. Bloino, B. G. Janesko, R. Gomperts, B. Mennucci, H. P. Hratchian, J. V. Ortiz, A. F. Izmaylov, J. L. Sonnenberg, D. Williams-Young, F. Ding, F. Lipparini, F. Egidi, J. Goings, B. Peng, A. Petrone, T. Henderson, D. Ranasinghe, V. G. Zakrzewski, J. Gao, N. Rega, G. Zheng, W. Liang, M. Hada, M. Ehara, K. Toyota, R. Fukuda, J. Hasegawa, M. Ishida, T. Nakajima, Y. Honda, O. Kitao, H. Nakai, T. Vreven, K. Throssell, J. A. Montgomery, Jr., J. E. Peralta, F. Ogliaro, M. J. Bearpark, J. J. Heyd, E. N. Brothers, K. N. Kudin, V. N. Staroverov, T. A. Keith, R. Kobayashi, J. Normand, K. Raghavachari, A. P. Rendell, J. C. Burant, S. S. Iyengar, J. Tomasi, M. Cossi, J. M. Millam, M. Klene, C. Adamo, R. Cammi, J. W. Ochterski, R. L. Martin, K. Morokuma, O. Farkas, J. B. Foresman, and D. J. Fox, Gaussian 16, Revision C.01, Gaussian, Inc., Wallingford CT, 2016.

[3] Y. Zhao, D. G. Truhlar, “The M06 suite of density functionals for main group thermochemistry, thermochemical kinetics, noncovalent interactions, excited states, and transition elements: two new functionals and systematic testing of four M06-class functionals and 12 other functionals” *Theor. Chem. Acc.* **2008**, *120*, 215–241.

[4] S. S. Leang, F. Zahariev, M. S. Gordon, “Benchmarking the performance of time-dependent density functional methods” *J. Chem. Phys.* **2012**, *136*, 104101.

[5] D. K. Roper, W. Ahn, M. Hoepfner, “Microscale Heat Transfer Transduced by Surface Plasmon Resonant Gold Nanoparticles” *J. Phys. Chem. C Nanomater. Interfaces* **2007**, *111*, 3636–3641.

[6] H. Xiang, L. Zhao, L. Yu, H. Chen, C. Wei, Y. Chen, Y. Zhao, “Self-assembled organic nanomedicine enables ultrastable photo-to-heat converting theranostics in the second near-infrared biowindow” *Nat. Commun.* **2021**, *12*, 218.

[7] H. Dai, Z. Cheng, T. Zhang, W. Wang, J. Shao, W. Wang, Y. Zhao, X. Dong, L. Zhong, “Boron difluoride formazanate dye for high-efficiency NIR-II fluorescence imaging-guided cancer photothermal therapy” *Chin. Chem. Lett.* **2022**, *33*, 2501–2506.

[8] H. Li, H. Dai, A. Mei, X. Ruan, W. Wang, D. Yang, W. Wang, Q. Zhang, X. Dong, J. Shao, “Triphenylamine flanked boron difluoride formazanate for NIR-II fluorescence imaging-guided photothermal therapy” *Dyes Pigments* **2022**, *205*, 110478.

[9] M. Yang, S. Wang, X. Ou, J. Ni, S. Segawa, J. Sun, F. Xu, R. T. K. Kwok, J. Zhao, J. W. Y. Lam, G. Jin, B. Z. Tang, “Reengineering of Donor–Acceptor–Donor Structured Near-Infrared II Aggregation-Induced Emission Luminogens for Starving-Photothermal Antitumor and Inhibition of Lung Metastasis” *ACS Nano* **2024**, *18*, 30069–30083.

[10] M. Peng, H. Wei, Q. Wang, J. Guan, M. Yin, “Naphthalimide Nanoprobe with Enhanced Electron-Withdrawing Ability and Large Stokes Shift for NIR-II Fluorescence Imaging-Guided Phototheranostics” *ACS Appl. Mater. Interfaces* **2025**, *17*, 11742–11751.

[11] H. Dai, J. Pan, J. Shao, K. Xu, X. Ruan, A. Mei, P. Chen, L. Qu, X. Dong, “Boosting Nonradiative Decay of Boron Difluoride Formazanate Dendrimers for NIR-II Photothermal Theranostics” *Angew. Chem. Int. Ed.* **2025**, *64*, e202503718.

[12] K. Xu, M. Luo, W. Wang, T. Zhang, Y. Chen, J. Shao, P. Chen, X. Dong, Y. Cai, “Boron Difluoride Formazanate Dye With Donor Planarization Engineering for 1060 nm Laser Activated Photothermal Theranostics” *Adv. Sci.* **2025**, *12*, e06226.

[13] H. Dai, J. Shao, D. Lei, K. Xu, T. Zhang, A. Mei, P. Chen, Y. Guo, X. Dong, “Suppressing Nonradiative Decay in BF2 Formazanates via Donor Bromination for Cancer Phototheranostics” *ACS Nano* **2025**, *19*, 35842–35852.
